# PDE4DIP contributes to colorectal cancer growth and chemoresistance through modulation of the NF1/RAS signaling axis

**DOI:** 10.1038/s41419-023-05885-y

**Published:** 2023-06-24

**Authors:** Rulu Pan, Juji Dai, Weicheng Liang, Hongxiao Wang, Lin Ye, Siqi Ye, Ziqi Lin, Shishun Huang, Yan Xiong, Li Zhang, Liting Lu, Ouchen Wang, Xian Shen, Wanqin Liao, Xincheng Lu

**Affiliations:** 1 School of Basic Medical Sciences, Wenzhou Medical University, Wenzhou, 325035 China; 2 Department of Colorectal and Anal Surgery, The First Affiliated Hospital of Wenzhou Medical University, Wenzhou, 325000 China; 3 Department of Breast Surgery, The First Affiliated Hospital of Wenzhou Medical University, Wenzhou, 325000 China; 4 Department of General Surgery, The First Affiliated Hospital of Wenzhou Medical University, Wenzhou, 325000 China

**Keywords:** Oncogenes, Colorectal cancer

## Abstract

Phosphodiesterase 4D interacting protein (PDE4DIP) is a centrosome/Golgi protein associated with cyclic nucleotide phosphodiesterases. PDE4DIP is commonly mutated in human cancers, and its alteration in mice leads to a predisposition to intestinal cancer. However, the biological function of PDE4DIP in human cancer remains obscure. Here, we report for the first time the oncogenic role of PDE4DIP in colorectal cancer (CRC) growth and adaptive MEK inhibitor (MEKi) resistance. We show that the expression of PDE4DIP is upregulated in CRC tissues and associated with the clinical characteristics and poor prognosis of CRC patients. Knockdown of PDE4DIP impairs the growth of KRAS-mutant CRC cells by inhibiting the core RAS signaling pathway. PDE4DIP plays an essential role in the full activation of oncogenic RAS/ERK signaling by suppressing the expression of the RAS GTPase-activating protein (RasGAP) neurofibromin (NF1). Mechanistically, PDE4DIP promotes the recruitment of PLCγ/PKCε to the Golgi apparatus, leading to constitutive activation of PKCε, which triggers the degradation of NF1. Upregulation of PDE4DIP results in adaptive MEKi resistance in KRAS-mutant CRC by reactivating the RAS/ERK pathway. Our work reveals a novel functional link between PDE4DIP and NF1/RAS signal transduction and suggests that targeting PDE4DIP is a promising therapeutic strategy for KRAS-mutant CRC.

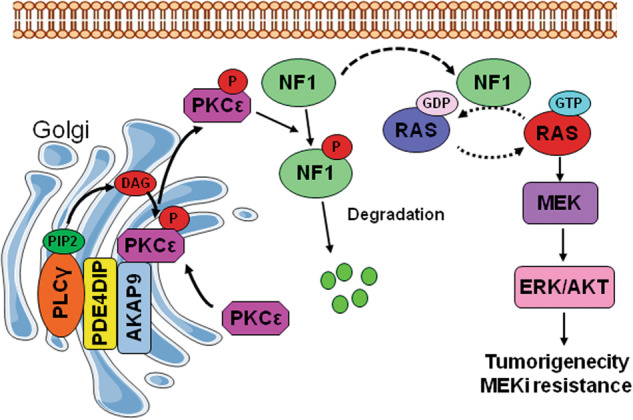

## Introduction

Colorectal cancer (CRC) is the third most common cancer worldwide, with approximately two million patients newly diagnosed with CRC annually [1]. CRC carcinogenesis is a multistep process resulting from the accumulation of genetic mutations and epigenetic modifications in colonic mucosal cells, ultimately leading to the initiation and malignant progression of CRC [2]. KRAS is the most commonly mutated oncogene in colon cancer patients, and there are no clinically proven strategies for the treatment of KRAS-driven CRC [3, 4]. Therefore, a more complete understanding of the regulation of this key gene will contribute to the clinical treatment of CRC.

Phosphodiesterase 4D interacting protein (PDE4DIP) is a CDK5RAP2 paralog in vertebrates [5]. In a yeast two-hybrid screen, PDE4DIP was identified as an interactor of phosphodiesterase 4D (PDE4D), an enzyme controlling the cAMP level [6]. With several transcript variants encoding different isoforms, PDE4DIP is ubiquitously expressed in mammalian cells, but its biological functions remain largely unknown [5–7]. PDE4DIP has been reported to play an important role in the regulation of cardiac contractility by affecting the phosphorylation of cMyBPC [8]. Two other studies showed that PDE4DIP variants are associated with an increased risk for ischemic stroke and that defects in this gene may be a cause of myeloproliferative disorders (MPDs) [9, 10]. In mammalian cells, PDE4DIP localizes to the centrosome and Golgi apparatus and binds directly to AKAP9, EB1/EB3, and FAM161A to form a functional complex [11–13]. Disrupting PDE4DIP expression affects endoplasmic reticulum (ER)-to-Golgi trafficking, Golgi/centrosome organization, and microtubule assembly [5, 6, 11]. Recently, accumulating evidence has implied that the PDE4DIP gene is associated with cancer risk [7]. For example, the survival of glioblastoma patients was found to be correlated with the PDE4DIP expression level [14]. A Sleeping Beauty transposon-mediated screen identified PDE4DIP as a susceptibility gene for adenomatous polyposis coli (Apc)-dependent intestinal tumorigenesis in mice [15]. PDE4DIP is a commonly mutated gene in leukemia, liver cancer, and nasopharyngeal carcinoma [16–19], and novel mutations have been reported in tumors originating in various tissues, including the lung, thyroid, ovary [20–22], kidney, peritoneum and bone marrow [23–25]. Genomic loss/gain and rearrangement of PDE4DIP have been discovered in prostate cancer, glioma, pineoblastoma, and intimal sarcoma [26–29]. Moreover, two recent studies demonstrated that loss of Mmg (an alternatively spliced isoform of the PDE4DIP gene) suppresses the growth of medulloblastoma and that depletion of another specific isoform of PDE4DIP leads to inhibition of cell proliferation and motility [30, 31]. Together, these findings suggest that PDE4DIP may play a critical role in cancer.

In this study, we investigated the functional roles of PDE4DIP in CRC. We found that PDE4DIP plays an essential role in the growth of KRAS-mutant CRC by promoting PKCε-mediated degradation of the Ras GTPase-activating protein (RasGAP) neurofibromin (NF1). We also identified PDE4DIP as a driver of adaptive resistance to MEK inhibitor (MEKi) therapy in KRAS-mutant CRC. Our study suggests that PDE4DIP is a critical upstream regulator of the growth and chemoresistance of KRAS-driven human CRC.

## Results

### PDE4DIP is highly expressed in human CRC

**Fig. 1:**
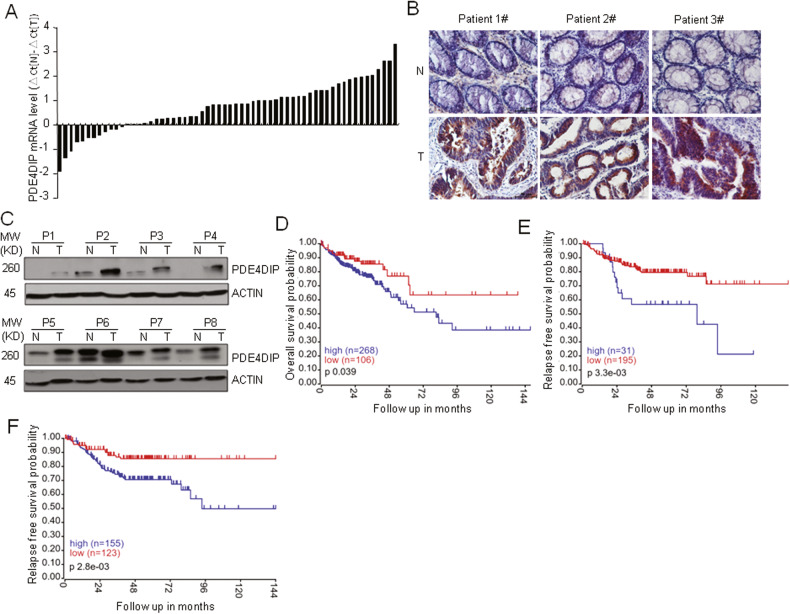
The expression of PDE4DIP is upregulated in human CRC tissues and associated with poor survival of patients. **A** qRT‒PCR analysis of PDE4DIP mRNA levels in human CRC tumors. PDE4DIP mRNA expression in 60 paired CRC tumors and adjacent normal mucosa was assessed by qRT‒PCR. **B** Immunohistochemical staining of the PDE4DIP protein in CRC specimens (T) and paired adjacent normal intestine samples (N). Scale bar, 50 μm. **C** Western blot showing the protein levels of PDE4DIP in CRC samples (T) and adjacent normal tissues (N) from eight patients. **D** Kaplan‒Meier survival analysis of CRC patients with PDE4DIP-high vs. PDE4DIP-low tumors from the TCGA cohort. Normalized TCGA data were downloaded from cBioPortal (http://www.cbioportal.org/, colorectal adenocarcinoma, PanCancer Atlas), and PDE4DIP mRNA expression levels in patients with colon adenocarcinoma were used for Kaplan‒Meier survival analysis. **E** Kaplan‒Meier analysis of relapse-free survival in CRC patients with PDE4DIP-high vs. PDE4DIP-low tumors from the GSE14333 cohort. **F** Kaplan‒Meier analysis of relapse-free survival in CRC patients from a combined cohort comprising GSE14333, GSE17536, and GSE17537. All Kaplan‒Meier curves were generated by the R2 program (https://hgserver1.amc.nl/cgi-bin/r2/main.cgi).

To explore the dysregulation of PDE4DIP in CRC, we analyzed PDE4DIP mRNA expression in primary human CRC tumor samples. In 48 of the 60 (80%) primary CRC tumors, the PDE4DIP transcript level was increased in tumor samples compared with the matched adjacent normal mucosa samples (Fig. 1A). The upregulation of PDE4DIP protein expression was also validated by Western blotting and immunohistochemical (IHC) staining (Fig. 1B, C). Correlation analysis of our CRC cohort revealed significant associations between upregulated expression of PDE4DIP and higher TNM stage, infiltration and lymph node metastasis (Table S1). To further determine the clinical significance of PDE4DIP, datasets from The Cancer Genome Atlas (TCGA) and Gene Expression Omnibus (GEO; GSE14333, GSE17536, and GSE17537) cohorts were mined for Kaplan‒Meier analysis. PDE4DIP expression was confidently correlated with patient prognosis in three independent CRC patient cohorts. Patients with a higher level of PDE4DIP expression had a shorter overall or relapse-free survival time than those with a lower level of PDE4DIP expression (Fig. 1D–F). Together, these results indicate that upregulation of PDE4DIP is associated with poor prognosis in CRC patients, suggesting that PDE4DIP may play an important role in CRC.

### PDE4DIP plays an oncogenic role in CRC growth

**Fig. 2:**
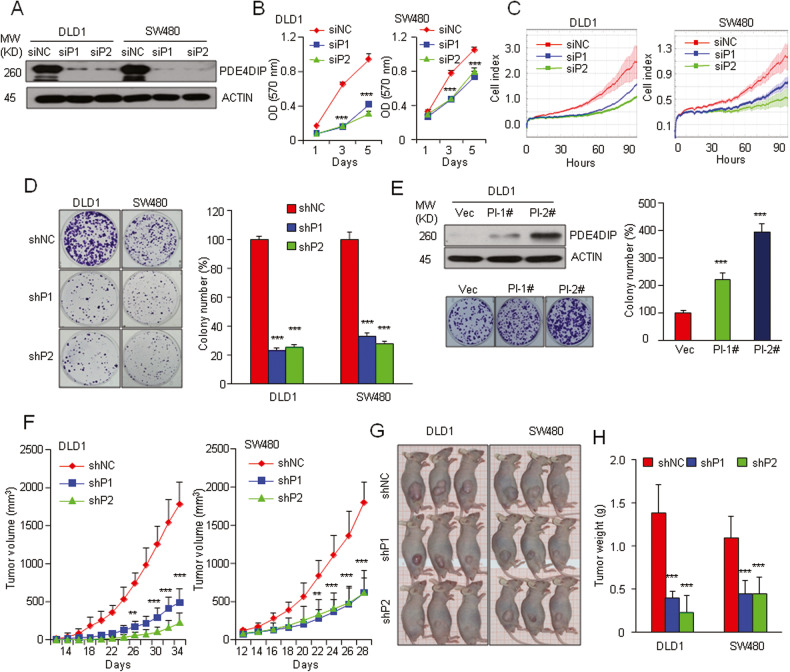
PDE4DIP promotes CRC cell proliferation in vitro and tumorigenesis in vivo. **A** siRNA-mediated knockdown of full-length PDE4DIP (isoform 1) expression was evaluated by Western blotting. DLD1 and SW480 cells were transfected with a negative control siRNA (siNC) or either of two siRNAs targeting PDE4DIP (siP1 and siP2) for 48 h. **B**, **C** MTT assay and RTCA in control and PDE4DIP-silenced DLD1 and SW480 cells. The data are representative of at least three independent experiments performed in triplicate. **D** Colony formation assay of control and PDE4DIP shRNA-transfected CRC cells. DLD1 and SW480 cells were infected with lentivirus containing control shRNA (shNC) or an shRNA targeting PDE4DIP (shP1, shP2). Left, representative images of the assay; right, data representative of at least three independent experiments performed in triplicate. **E** Colony formation assay of control and DLD1 cells stably transfected with PDE4DIP. A control clone transfected with empty vector (Vec) and two randomly selected stable clones with medium or high expression of full-length PDE4DIP (PI-1# and PI-2#, respectively) were used in the assay. Left, Western blot showing PDE4DIP (Myc-tagged) levels in DLD1 cells (top) and representative images of the assay (bottom); right, data representative of at least three independent experiments performed in triplicate. **F** Growth curve of xenografts generated from control and PDE4DIP shRNA-transfected CRC cells. **G** Representative images of half of the animals in the control and PDE4DIP shRNA-transfected groups are shown at 34 (DLD1) and 28 (SW480) days after injection, depicting the extent of tumor burden. **H** Weights of CRC xenografts derived from control and PD4DIP shRNA-transfected CRC cells (*n* = 6 mice). **B, D, E, H** the data are presented as the means ± SDs, and the *P* values were determined by Student’s *t* test. **F** The data are presented as the means ± SEMs (*n* = 6 mice), and the *P* values were determined by one-way ANOVA. **P < 0.01, ***P < 0.001 vs. control.

To explore the biological role of PDE4DIP in CRC, we chose DLD1 and SW480 cells, both of which express relatively high levels of full-length PDE4DIP (PI) and isoform 5 (PI-5), for subsequent studies (Fig. S1A). Knockdown of PDE4DIP by two independent siRNAs (siP1 and siP2) significantly reduced the proliferation capacity of CRC cells, as determined by both an MTT assay and real-time cell analysis (RTCA) (Fig. 2A–C). Transfection of two shRNAs (shP1 and shP2) targeting PDE4DIP also resulted in a decrease of more than 65% in the cell colony-forming capacity (Fig. 2D, Fig. S1B). In contrast, stable overexpression of full-length PDE4DIP in DLD1 cells significantly increased the colony-forming capacity of tumor cells (Fig. 2E). Notably, specific knockdown or overexpression of PDE4DIP isoform 5 in these two CRC cell lines did not affect their proliferation (Fig. S1C, D). These results indicate that full-length PDE4DIP functions as an enhancer of CRC cell proliferation in vitro. To assess the effect of PDE4DIP on tumor growth in vivo, we subcutaneously injected DLD1 and SW480 cells stably transduced with lentiviral vectors harboring scrambled control shRNA (shNC) or PDE4DIP shRNA into nude mice and then monitored the growth of the resultant tumors. As shown in Fig. 2F–H, tumor growth in the PDE4DIP-silenced groups (shP1 and shP2) was hindered and significantly reduced compared with that in the control group, as revealed by subsequent analysis of tumor volume and final tumor weight. Taken together, the in vitro and in vivo results demonstrate that PDE4DIP plays an oncogenic role in CRC growth.

### PDE4DIP is required for the full activation of oncogenic RAS signaling

**Fig. 3:**
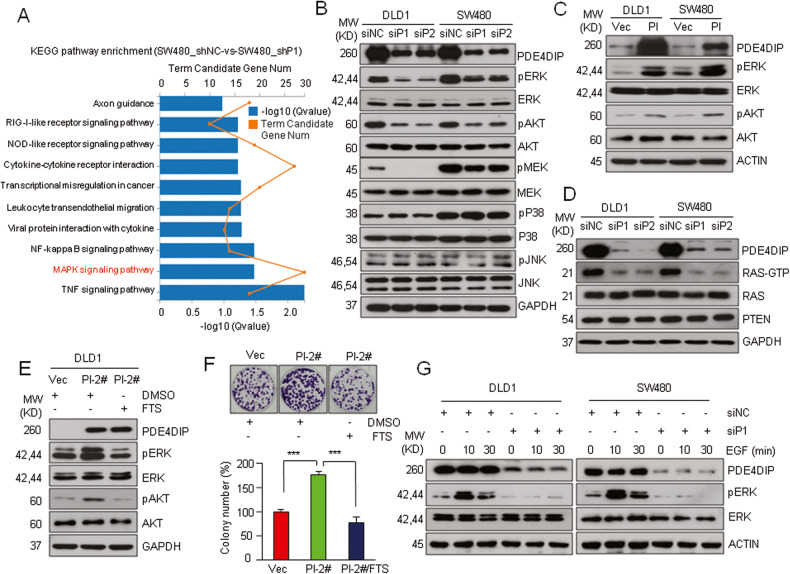
PDE4DIP promotes CRC growth via activation of RAS/ERK/AKT signaling. **A** KEGG enrichment analysis of the pathways in PDE4DIP-silenced SW480 cells. Total RNA isolated from SW480 cells infected with lentivirus containing control shRNA (shNC) or shRNA targeting PDE4DIP (shP1) was subjected to RNA-seq. **B** Western blot analysis was performed to determine the activation of MAPK/AKT signaling in control (siNC) and PDE4DIP-silenced (siP1, siP2) CRC cells. **C** Western blot showing the activation of ERK/AKT in PDE4DIP-overexpressing CRC cells. DLD1 and SW480 cells were transfected with empty vector (Vec) or the Myc-tagged PDE4DIP expression plasmid (PI) for 48 h. **D** A RAS GST-RBD pulldown assay was performed to determine RAS activity in control and PDE4DIP-silenced CRC cells. **E** Western blot showing that the RAS inhibitor FTS (salirasib) attenuated PDE4DIP-promoted ERK/AKT phosphorylation. DLD1 cells stably transfected with empty vector (Vec) or the PDE4DIP plasmid (PI-2) were treated with DMSO or FTS (75 μM) for 72 h. **F** Colony formation assay showing that FTS treatment impaired PDE4DIP-promoted DLD1 cell proliferation. Top, representative images of the assay; bottom, data representative of at least three independent experiments performed in triplicate. The data are presented as the means ± SDs, and the *P* values were determined by Student’s *t* test. ***P < 0.001. **G** Western blot showing that presilencing PDE4DIP blocked EGF-stimulated ERK activation. DLD1 and SW480 cells transfected with siNC or with siP1 targeting PDE4DIP were serum starved for 24 h prior to stimulation with 100 ng/ml EGF for the indicated times.

Next, we sought to investigate the signaling pathways involved in PDE4DIP-induced CRC tumorigenesis. Since PDE4DIP has been considered to interact with PDE4D, a protein modulating the cAMP level and PKA activity [6, 8], we first hypothesized that PDE4DIP may affect CRC cell growth via the PKA signaling pathway. However, knockdown of PDE4DIP in DLD1 and SW480 cells did not affect the expression of PDE4D or its downstream PKA signaling effectors (Fig. S2A). Immunofluorescence imaging showed that PDE4DIP was a Golgi-associated protein that colocalized with GM130 (Fig. S2B), whereas PDE4D was diffusely expressed throughout the cells, and no colocalization of PDE4DIP and PDE4D was observed (Fig. S2C). These findings suggest that PDE4DIP promotes CRC development through a PDE4D/PKA-independent mechanism. Gene Ontology (GO) and Kyoto Encyclopedia of Genes and Genomes (KEGG) enrichment analyses showed that tumor growth- and cell survival-related pathways (MAPK and GTPase activity) were predominant among the downregulated pathways in PDE4DIP-silenced cells (Fig. 3A, Fig. S2D). Gene set enrichment analysis (GSEA) of the TCGA dataset and two other GEO cohorts also revealed that the gene sets related to MAPK and mTOR/AKT signaling pathways were enriched in tumor samples with high PDE4DIP expression (Fig. S3A–C). Thus, we investigated whether MAPK or mTOR/AKT signaling pathway plays a role in PDE4DIP-induced CRC growth. Consistent with the bioinformatics analysis data, knockdown of PDE4DIP in DLD1 and SW480 cells led to significant reductions in MEK, ERK, and AKT phosphorylation without altering the abundances of the corresponding proteins (Fig. 3B). In contrast, ectopic overexpression of full-length PDE4DIP enhanced the phosphorylation of ERK and AKT in these two CRC cell lines (Fig. 3C). Moreover, pretreatment with MEK or AKT inhibitors attenuated PDE4DIP-promoted tumor cell proliferation (Fig. S4A). These results suggest that MAPK/AKT signal activation is responsible for PDE4DIP-promoted CRC tumor growth. Both DLD1 and SW480 cells harbor mutationally activated KRAS proteins. In the GST-RBD pulldown assay, PDE4DIP-silenced DLD1 and SW480 cells exhibited lower RAS-GTP levels than their siNC counterparts (Fig. 3D). Moreover, pretreatment with the RAS inhibitor salirasib (S-farnesylthiosalicylic acid, FTS) not only blocked PDE4DIP-promoted ERK/AKT phosphorylation but also nullified the promoting effect of PDE4DIP on tumor cell growth (Fig. 3E, F, Fig. S4B). In addition, silencing PDE4DIP in DLD1 and SW480 cells completely blocked EGF-stimulated ERK activation (Fig. 3G). These findings suggest that PDE4DIP promotes CRC tumor growth via the RAS/MAPK/AKT axis and that PDE4DIP plays an essential role in the full activation of RAS signaling in KRAS-mutant CRC cells. In addition, TCGA database analysis revealed a significant association between high PDE4DIP expression and a poor outcome in CRC patients harboring KRAS mutations but not in those lacking KRAS mutations (Fig. S4C). These results further support the idea that PDE4DIP plays an important role in the progression of KRAS-mutant CRC.

### PDE4DIP promotes overactivation of RAS signaling via suppression of NF1 expression

**Fig. 4:**
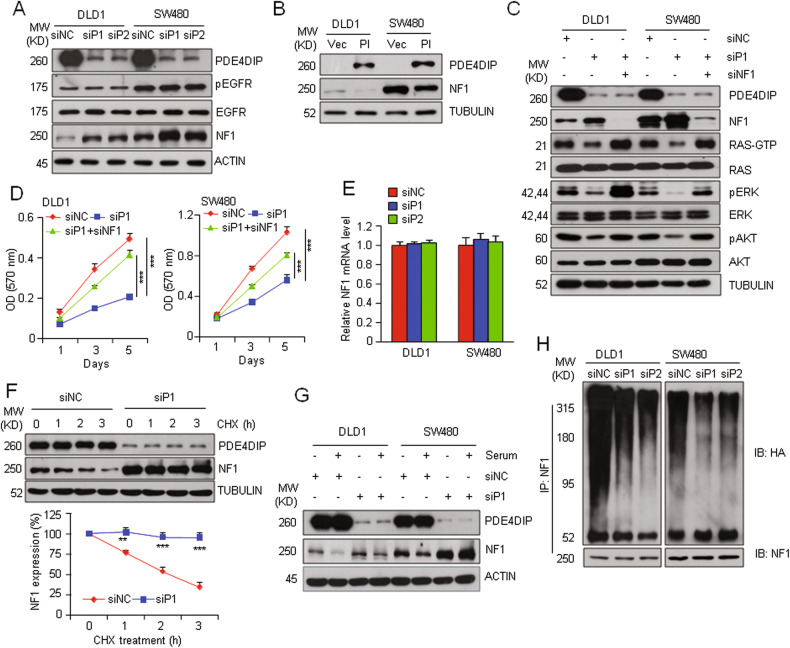
PDE4DIP enhances oncogenic RAS signaling via downregulation of NF1. **A** Western blot analysis of the levels of phosphorylated EGFR (pEGFR), EGFR, and NF1 in control (siNC) and PDE4DIP-silenced (siP1, siP2) CRC cells. **B** Western blot analysis of NF1 expression in PDE4DIP-overexpressing CRC cells. DLD1 and SW480 cells were transfected with empty vector (Vec) or the Myc-tagged PDE4DIP expression plasmid (PI) for 48 h. **C** Western blot showing that presilencing of NF1 (siNF1) restored the activation of RAS/ERK/AKT signaling in PDE4DIP-silenced (siP1) CRC cells. **D** MTT assay showing that presilencing of NF1 (siNF1) attenuated the inhibitory effect of PDE4DIP interference on cell proliferation. The data are representative of at least three independent experiments performed in triplicate and are presented as the means ± SDs. **E** qRT‒PCR analysis of NF1 transcript levels in control and PDE4DIP-silenced CRC cells. **F** Cycloheximide (CHX) chase assay to determine the degradation of NF1 in control (siNC) and PDE4DIP-silenced (siP1) CRC cells. Cells were treated with 100 μg/ml cycloheximide for the indicated times. The band intensity of NF1 was normalized to that of α-tubulin, and the intensity at time zero was set as 100%. Top, representative Western blot images; bottom, data presented as the mean ± SD of three biological replicates. **G** Western blot analysis of NF1 levels in control and PDE4DIP-silenced CRC cells. Cells were starved for 12 h and stimulated with medium containing 10% FBS for 10 min. **H** Western blot analysis of NF1 ubiquitination in control and PDE4DIP-silenced CRC cells. **D, F** the *P* values were determined by Student’s *t* test. **P < 0.01, ***P < 0.001 vs. control.

Having found that silencing PDE4DIP effectively suppressed RAS/MAPK/AKT signaling in KRAS-mutant CRC cells, we aimed to define the underlying mechanism. Silencing of PDE4DIP in DLD1 and SW480 cells did not affect the activation of EGFR, FAK, and SRC, nor did it change the expression of p120GAP, but resulted in a significant increase in the protein level of NF1 (Fig. 4A, Fig. S5A), another well-established GAP family member that negatively regulates RAS activation [32, 33]. Conversely, overexpressing PDE4DIP in these two CRC cell lines reduced the NF1 protein expression level (Fig. 4B). These results prompted us to investigate the role of NF1 in PDE4DIP-promoted RAS activation. Silencing NF1 in DLD1 and SW480 cells enhanced the activation of RAS/ERK/AKT signaling (Fig. S5B). Conversely, ectopic overexpression of the NF1 GAP-related domain (NF1-GRD) markedly impaired RAS activity and the growth of these two KRAS-mutant CRC cell lines (Fig. S5C, D). More importantly, a rescue experiment showed that the inhibitory effect of PDE4DIP interference on RAS/ERK/AKT signaling and cell proliferation was significantly attenuated by simultaneous transfection of siRNA targeting NF1. (Fig. 4C, D, Fig. S5E). Together, these results suggest that downregulation of NF1 plays an essential role in PDE4DIP-promoted overactivation of RAS/ERK/AKT signaling and CRC tumor growth. Silencing PDE4DIP had little effect on the mRNA level of NF1 in DLD1 and SW480 cells, nor changed NF1 mRNA stability (Fig. 4E, Fig. S5F). A cycloheximide (CHX) chase assay showed that silencing PDE4DIP delayed the degradation of the NF1 protein (Fig. 4F). These findings suggest that PDE4DIP downregulates NF1 expression at the posttranslational level. Silencing PDE4DIP obviously suppressed serum-induced degradation of NF1 (Fig. 4G). Moreover, silencing PDE4DIP expression significantly decreased the accumulation of ubiquitinated NF1 in DLD1 and SW480 cells (Fig. 4H). Together, these results suggest that PDE4DIP suppresses the expression of NF1 in CRC cells by promoting its ubiquitination and degradation.

### PDE4DIP promotes the degradation of NF1 via activation of PKCε

**Fig. 5:**
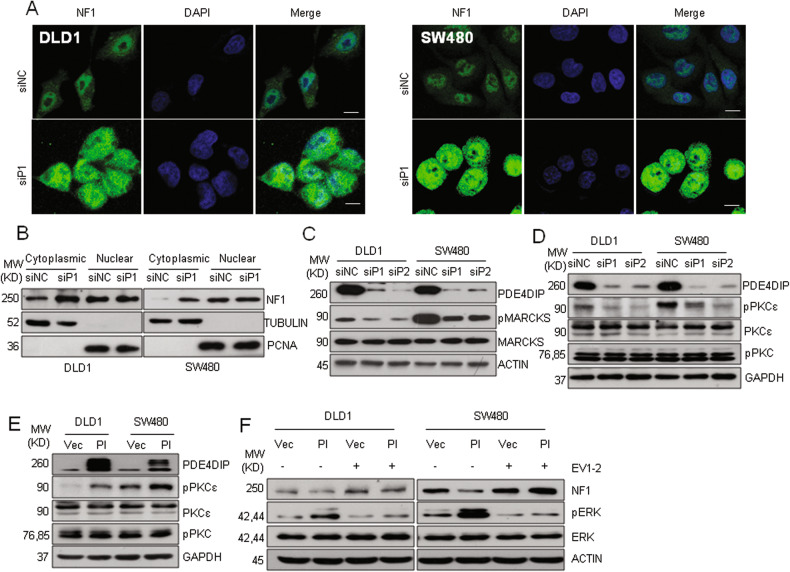
PDE4DIP promotes NF1 degradation through activation of PKCε. **A** Immunofluorescence staining showing the subcellular localization of NF1 in control (siNC) and PDE4DIP-silenced (siP1) CRC cells. Scale bar, 10 μm. **B** Western blot analysis was performed to determine the cytoplasmic and nuclear NF1 levels in control (siNC) and PDE4DIP-silenced (siP1) CRC cells. **C** Western blot analysis of the MARCKS and phosphorylated MARCKS (pMARCKS) levels in control (siNC) and PDE4DIP-silenced (siP1, siP2) CRC cells. **D** Western blot analysis of the total PKCε, phosphorylated PKCε (pPKCε) and phosphorylated pan-PKC (pPKC) levels in control and PDE4DIP-silenced CRC cells. **E** Western blot analysis of the activation of PKC in PDE4DIP-overexpressing CRC cells. DLD1 and SW480 cells were transfected with empty vector (Vec) or the Myc-tagged PDE4DIP expression plasmid (PI) for 48 h. **F** Western blot showing that the PKCε inhibitor EV1-2 blocked the PDE4DIP-promoted reduction in NF1 and phosphorylation of ERK. Cells transfected with empty vector (Vec) or the PDE4DIP expression plasmid (PI) were exposed to DMSO or EV1-2 (1.0 μM) for 30 min.

Next, we explored the mechanism of how PDE4DIP modulates the stability of NF1 protein. Silencing PDE4DIP in DLD1 and SW480 cells significantly increased the accumulation of NF1 in the cytoplasm but did not change its abundance in the nucleus (Fig. 5A, B). In neural cells, activated PKC has been shown to facilitate the phosphorylation and rapid proteasomal degradation of NF1 in the cytoplasm [34, 35]. In DLD1 and SW480 cells, knocking down PDE4DIP expression resulted in a marked reduction in the level of phosphorylated MARCKS, a well-characterized PKC substrate, whereas overexpression of PDE4DIP increased the level of phosphorylated MARCKS (Fig. 5C, Fig. S6A). Treatment with the PKC inhibitor RO-31-8220 also resulted in obvious NF1 accumulation in both CRC cell lines (Fig. S6B). These results suggest that PKC may function in PDE4DIP-induced NF1 degradation in CRC cells. Knockdown of PDE4DIP in DLD1 and SW480 cells decreased the level of phosphorylated PKCε, whereas ectopic overexpression of PDE4DIP markedly enhanced PKCε phosphorylation (Fig. 5D, E). Moreover, treatment with the PKCε inhibitor EV1-2 not only blocked the PDE4DIP overexpression-induced reduction in the NF1 level but also inhibited PDE4DIP-promoted ERK activation (Fig. 5F). In addition, blocking PKCε activation alone resulted in effects similar to those of interfering with PDE4DIP, including suppression of NF1 degradation and ERK activation (Fig. S6C–F). Knockdown of PKCε expression in DLD1 and SW480 cells also resulted in reduced cell proliferation (Fig. S6G). Taken together, these data indicate that PKCε activation plays an essential role in PDE4DIP-promoted NF1 degradation and CRC tumor growth.

### PDE4DIP promotes the enrichment and activation of PKCε in the Golgi apparatus

**Fig. 6:**
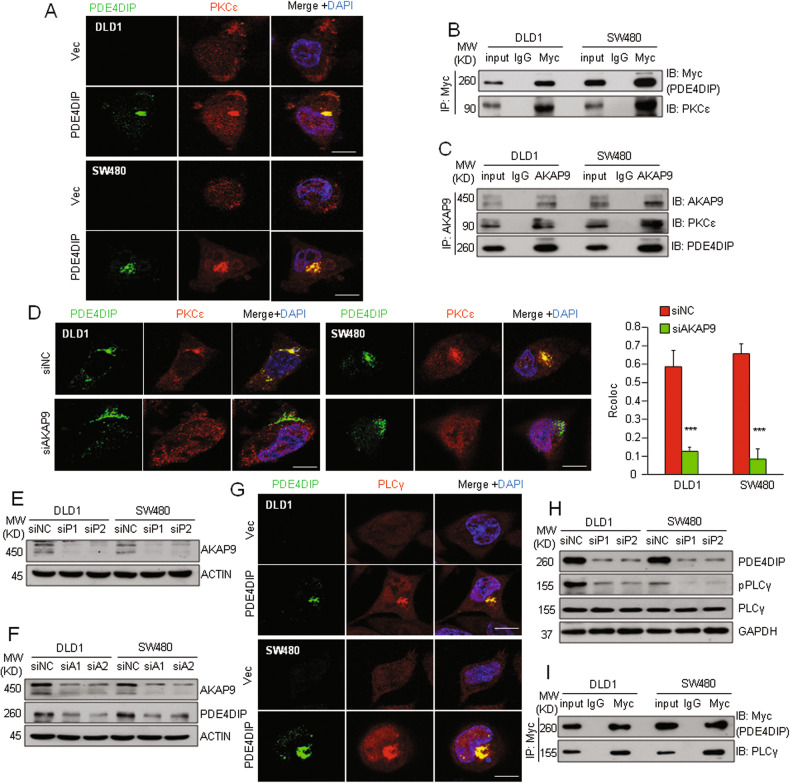
PDE4DIP promotes the recruitment of PLCγ/PKCε to the Golgi apparatus. **A** Immunofluorescence staining showing the colocalization of PDE4DIP (green) and PKCε (red) at the Golgi apparatus. Cells were transfected with empty vector (Vec) or the PDE4DIP expression plasmid (PI) for 48 h. Scale bars, 10 μm. **B** Coimmunoprecipitation of PDE4DIP with PKCε in DLD1 and SW480 cells. Cells were transfected with the Myc-tagged PDE4DIP expression plasmid, and cell lysates were immunoprecipitated with an anti-Myc antibody and subjected to Western blotting with anti-Myc and anti-PKCε antibodies. **C** Coimmunoprecipitation of endogenous AKAP9 with PDE4DIP or PKCε in DLD1 and SW480 cells. Cell lysates were immunoprecipitated with an anti-AKAP9 antibody and subjected to Western blotting with anti-AKAP9, anti-PDE4DIP, and anti-PKCε antibodies. **D** Immunofluorescence analysis of the level of PDE4DIP (green) and PKCε (red) colocalization at the Golgi apparatus in control (siNC) and AKAP9-silenced (siAKAP9) CRC cells. Scale bars, 10 μm. Representative images (left) and colocalization values (Rcoloc) of PDE4DIP and PKCε are shown (right). The data are presented as the means ± SDs of three biological replicates, and the *P* values were determined by Student’s *t* test. ***P < 0.001 vs. control. **E** Western blot analysis of AKAP9 expression in control and PDE4DIP-silenced CRC cells. **F** Western blot analysis of PDE4DIP expression in control and AKAP9-silenced CRC cells. Cells were transfected with control siNC or siRNA targeting AKAP9 (siA1, siA2) for 48 h. **G** Immunofluorescence staining showing the colocalization of PDE4DIP (green) and PLCγ (red) at the Golgi apparatus in CRC cells. Scale bars, 10 μm. **H** Western blot analysis of total PLCγ and phosphorylated PLCγ (pPLCγ) in control and PDE4DIP-silenced cells. **I** Coimmunoprecipitation of PDE4DIP with endogenous PLCγ in DLD1 and SW480 cells. Cell lysates were immunoprecipitated with an anti-Myc antibody and subjected to Western blotting with anti-Myc and anti-PLCγ antibodies.

After establishing that PDE4DIP and PKCε cooperate to downregulate NF1, we next sought to understand how PDE4DIP promotes PKCε activation. In PDE4DIP-overexpressing DLD1 and SW480 cells, we observed colocalization of PDE4DIP and PKCε as well as obvious enrichment of PKCε on the Golgi apparatus (Fig. 6A). The coimmunoprecipitation (Co-IP) assay demonstrated that PDE4DIP and PKCε were specifically coimmunoprecipitated (Fig. 6B). These findings suggest that the interaction between PDE4DIP and PKCε may promote the recruitment of PKCε to the Golgi apparatus. Previous studies reported that AKAP9 can anchor hypophosphorylated PKCε to the Golgi apparatus for dynamic autophosphorylation [36]. In DLD1 and SW480 cells, AKAP9 coimmunoprecipitated with both the PDE4DIP and PKCε proteins (Fig. 6C, Fig. S7A). The abundance of PDE4DIP-colocalized PKCε on the Golgi was decreased following AKAP9 knockdown (Fig. 6D). Moreover, knockdown of PDE4DIP expression led to a marked reduction in the protein level of AKAP9, and vice versa (Fig. 6E, F). These findings suggest that the expression of these two proteins is mutually dependent and that the interaction between PDE4DIP and AKAP9 is necessary for the recruitment of PKCε to the Golgi for autoactivation. Moreover, in PDE4DIP-overexpressing DLD1 and SW480 cells, we observed obvious enrichment of PLCγ on the Golgi apparatus (Fig. 6G). Furthermore, in both CRC cell lines, knockdown of PDE4DIP decreased the level of phosphorylated PLCγ, whereas ectopic overexpression of PDE4DIP markedly increased PLCγ phosphorylation (Fig. 6H, Fig. S7B). PDE4DIP and endogenous PLCγ also specifically coimmunoprecipitated (Fig. 6I). Taken together, these results suggest that PDE4DIP promotes the activation and distribution of PLCγ on the Golgi apparatus, which can further induce PKCε activation.

### Upregulation of PDE4DIP confers adaptive MEKi resistance

**Fig. 7:**
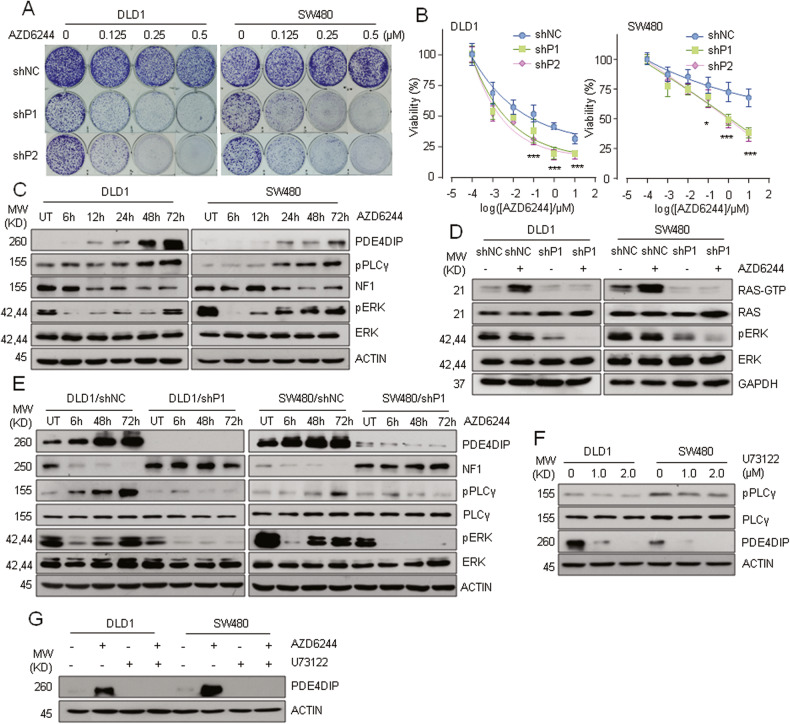
Knockdown of PDE4DIP abrogates adaptive MEKi resistance in CRC cells. **A** Colony formation assay of control and PDE4DIP-silenced DLD1 and SW480 cells treated with a MEKi. Cells infected with lentivirus containing control shRNA (shNC) or shRNAs targeting PDE4DIP (shP1, shP2) were treated with the indicated concentrations of AZD6244 for 2 weeks. The images are representative of at least three independent experiments. **B** Dose‒response curves of control and PDE4DIP-silenced DLD1 and SW480 cells treated with increasing concentrations of AZD6244 for 72 h. The data are representative at least three independent experiments performed in triplicate. The lines show the fitted curves, where each dot indicates the mean value of three technical replicates. The data are presented as the means ± SDs, and the *P* values were determined by Student’s *t* test. ***P < 0.001 vs. control. **C** Western blot analysis of NF1 levels and activation of PLCγ and ERK in AZD6244-resistant CRC cells. DLD1 and SW480 cells treated with AZD6244 (1.0 μM) were collected for lysis at the indicated time points. UT, untreated. **D** Western blot analysis of RAS activity and ERK phosphorylation in control and PDE4DIP-silenced cells following MEK inhibition. Cells infected with lentivirus containing control shRNA (shNC) or shRNA targeting PDE4DIP (shP1) were treated with DMSO or AZD6244 (1.0 μM) for 72 h. **E** Western blot showing that knockdown of PDE4DIP abolished the MEKi-induced reductions in NF1 expression and reactivation of PLCγ/ERK. Cells were treated with AZD6244 (1.0 μM) for the indicated times. UT, untreated. **F** Western blot analysis of PDE4DIP expression in CRC cells following PLCγ inhibition. Cells were treated with DMSO or U73122 (1.0 and 2.0 μM) for 24 h. **G** Western blot showing that inhibition of PLCγ blocked MEKi-induced PDE4DIP overexpression in CRC cells. Cells were treated with AZD6244 (1.0 μM) for 72 h and/or U73122 (1.0 μM) for 24 h. DMSO served as a control.

Currently, there is no effective therapy for the treatment of KRAS-mutant cancers [37]. MEKi treatment has shown convincing efficacy in preclinical and clinical studies, but it is also prone to induce adaptive resistance [38]. In the colony formation assay, both DLD1 and SW480 cells showed adaptive resistance to the MEKi AZD6244 (Fig. 7A), and knockdown of PDE4DIP restored the susceptibility of tumor cells to AZD6244 treatment (Fig. 7B). Significantly increased sensitivity was also observed in PDE4DIP-silenced CRC cells treated with another MEKi, trametinib (Fig. S8A, B). These results suggest that PDE4DIP contributes to adaptive MEKi resistance in CRC. After MEK inhibition, MEKi-resistant CRC cells showed an initial reduction in the level of phosphorylated ERK, which was restored within 72 h, accompanied by an increased RAS-GTP level (Fig. 7C, D). Silencing PDE4DIP blocked the increase in the RAS-GTP level resulting from chronic MEK inhibition in DLD1 and SW480 cells (Fig. 7D), and combined PDE4DIP interference and MEK inhibition synergistically inhibited the rebound of ERK signaling (Fig. 7D, E). These findings suggest that PDE4DIP is involved in MEKi-induced RAS overactivation and ERK reactivation. In AZD6244-treated DLD1 and SW480 cells, we observed a sustained increase in PDE4DIP protein expression and PLCγ phosphorylation, accompanied by decreased NF1 expression, and presilencing PDE4DIP completely abolished the AZD6244-induced activation of PLCγ and reduction in NF1 expression (Fig. 7C, E). In addition, combined PLCγ and MEK inhibition blocked the AZD6244-induced reduction in NF1 expression and reactivation of ERK and simultaneously restored the susceptibility of tumor cells to AZD6244 treatment (Fig. S8C, D). These findings suggest that upregulation of PDE4DIP confers adaptive MEKi resistance on KRAS-mutant CRC cells by overactivating PLCγ/RAS signaling. Intriguingly, PDE4DIP expression was dose-dependently inhibited by the PLCγ inhibitor U73122 (Fig. 7F), and inhibition of PLCγ blocked MEKi-induced PDE4DIP overexpression (Fig. 7G). Combined with the promoting effect of PDE4DIP on PLCγ activation, our results suggest the formation of a positive feedback loop between PDE4DIP and PLCγ, which accelerates the development of adaptive MEKi resistance in CRC cells.

## Discussion

**Fig. 8:**
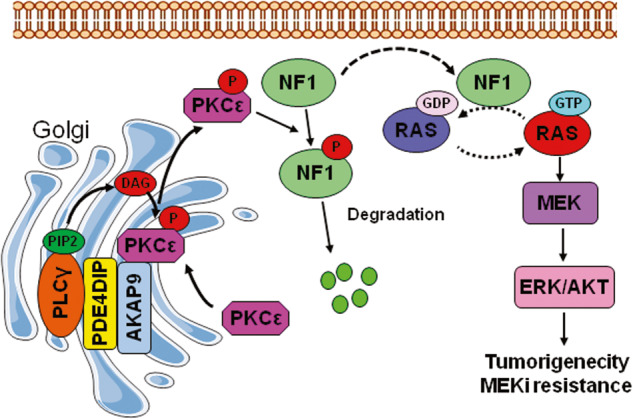
Schematic depiction of the mechanism by which PDE4DIP mediates RAS signaling activation in CRC cells. In CRC cells expressing high levels of PDE4DIP, binding of PDE4DIP and AKAP9 to the Golgi apparatus promotes the recruitment of PLCγ/PKCε to the Golgi for PKCε autoactivation, resulting in the release of phosphorylated PKCε to phosphorylate NF1 and target it for ubiquitin-mediated degradation and thus removing the inhibitory effect of NF1 on oncogenic RAS signal transduction.

Mutations in the PDE4DIP gene are common in cancers and are thought to be associated with cancer risk [15]. However, the functional roles of PDE4DIP in human cancer remain unknown. In this study, we present evidence that PDE4DIP amplifies ERK/AKT signaling through the core RAS pathway, thus supporting the proliferation of KRAS-mutant CRC cells. Mechanistically, PDE4DIP promotes the recruitment of PKCε to the Golgi for dynamic autoactivation, which enhances the ubiquitination and degradation of NF1 and thus removes the inhibitory effect of NF1 on oncogenic RAS signaling. PDE4DIP-promoted overactivation of RAS signaling also confers adaptive MEKi resistance on KRAS-mutant CRC cells. Based on our study, we propose a novel PDE4DIP-mediated RAS signal transduction mechanism that plays an important role in CRC tumorigenesis and chemoresistance (Fig. 8).

PDE4DIP has been described as a PDE4D-interacting protein that may regulate cAMP hydrolysis and PKA activation [6]. In medulloblastoma cells, PDE4DIP loss has been shown to mislocalize PDE4D3 from the centrosome, leading to local PKA overactivation [30]. In the current study, we found that PDE4DIP did not affect the activation of the PKA pathway in CRC cells. Our data indicated that the core RAS signaling pathway is responsible for PDE4DIP-promoted CRC progression. Mutation of the proto-oncogene KRAS is the most prominent oncogenic mutation in CRC, occurring in 30–40% of CRC patients [4]. RAS mutation triggers constitutive activation of downstream effectors such as MAPK and PI3K cascades to promote the malignant transformation and progression of CRC and confers resistance to anti-EGFR antibodies [4, 39]. In this study, we showed that knockdown of PDE4DIP impaired KRAS-mutant CRC tumor growth through suppression of oncogenic RAS/ERK/AKT signaling. Our findings support the hypothesis that PDE4DIP is required for maintaining the full activation of oncogenic RAS/ERK/AKT signaling and thus may play an essential role in KRAS-driven CRC progression. Clinically, inhibition of the KRAS oncoprotein has proven difficult. KRAS-mutant tumors are also insensitive to inhibition of upstream growth factor receptor signaling, and targeting downstream effectors such as MEK has been hampered by the activation of compensatory resistance mechanisms [37, 38, 40]. However, our study suggests that this independence may not be absolute. We demonstrated that inhibition of PDE4DIP is an unexpected vulnerability of KRAS-mutant CRC cells, suggesting that PDE4DIP may be a promising target for the effective treatment of KRAS-mutant CRC clinically.

Another important finding of our study is that PDE4DIP affects KRAS-mutant CRC tumor growth via PKCε-mediated destabilization of NF1. As an important RasGAP, NF1 functions as a tumor suppressor in multiple tumors, and loss of NF1 is common and associated with tumor growth as well as resistance to targeted therapy in CRC [41–43]. In the context of KRAS-mutant cancer, NF1 loss usually indicates overactivation of RAS and enhanced cell growth [44]. Our results demonstrated that PDE4DIP negatively regulates the protein level of NF1 in CRC cells. Moreover, we showed that interference with NF1 attenuated the inhibitory effects of PDE4DIP silencing on oncogenic RAS activity and cell proliferation. Our findings suggest that NF1 is the downstream transducer of PDE4DIP signaling and that the growth advantage of tumor cells with high PDE4DIP expression is most likely conferred by overactivation of oncogenic RAS signaling due to loss of NF1. Alterations in the NF1 protein level can be caused by various mechanisms, including gene mutation, DNA methylation, and ubiquitination [43, 44]. In this study, we demonstrated that PDE4DIP decreased the NF1 protein level in CRC cells by promoting its ubiquitination and degradation and that this decrease was associated with the activation of PKCε. PKCε is the only PKC isozyme that has been associated with oncogenesis [45]. Overexpression or activation of PKCε occurs in numerous cancers and has been shown to be necessary for KRAS-driven lung tumorigenesis [46, 47]. PKCε has been reported to collaborate with RAS/MAPK to initiate malignant transformation and tumor progression, but the mechanism by which oncogenic PKCε promotes RAS/MAPK activation is incompletely defined [46]. Here, we demonstrated that PDE4DIP-mediated activation of PKCε promotes the degradation of NF1 in CRC cells, resulting in overactivation of the downstream RAS/MAPK pathway. These findings suggest that altering the stability of NF1 may be a novel mechanism by which PKCε regulates RAS/MAPK signaling. In glioblastoma cells, PKCε has been shown to phosphorylate NF1 at Ser2808, thereby altering its nuclear distribution [48]. In glioma, another PKC family protein, PKCα, has been shown to phosphorylate NF1 in the cysteine/serine-rich domain, thereby promoting the ubiquitination and degradation of NF1 [34]. We found that in CRC cells, neither depletion of PDE4DIP nor blockade of PKCε activity altered the amount of NF1 in the nucleus, but whether PDE4DIP-mediated activation of PKCε promotes NF1 degradation in a PKCα-like phosphorylation pattern needs further study.

In this study, we found that PDE4DIP promotes the sustained activation of PKCε in CRC cells. PDE4DIP is a Golgi-associated protein and localizes predominantly to cis-Golgi networks by interacting with AKAP9 [6]. A previous study demonstrated that APAP9 can anchor hypophosphorylated PKCε in the Golgi area and serve as a scaffold for PKCε phosphorylation, and phosphorylated PKCε dynamically dissociates and is released into the cytosol [36]. We found that in CRC cells, PDE4DIP bound to both the AKAP9 and PKCε proteins and that interference with AKAP9 abolished the PDE4DIP-promoted distribution of PKCε on the Golgi apparatus. These findings suggest that PDE4DIP promotes the recruitment of PKCε to the Golgi apparatus in an AKAP9-dependent manner. Furthermore, we demonstrated that the stability of PDE4DIP and AKAP9 was mutually dependent. Our results suggest that the formation of the PDE4DIP/AKAP9 complex on the Golgi provides a stable docking platform for PKCε recruitment, while the diacylglycerol (DAG)-rich Golgi membrane may supply a finely tuned microenvironment for PKCε autophosphorylation [49]. Intriguingly, we found that PDE4DIP also promoted PLCγ activation and simultaneously increased its distribution on the Golgi, which could provide a sustained impetus for PKCε activation. Taken together, our findings support the hypothesis that PDE4DIP plays an essential role in the sustained activation of PKCε on the Golgi. In CRC cells, the PDE4DIP/AKAP9 complex recruits nonphosphorylated PKCε to the Golgi apparatus for its dynamic autophosphorylation. PDE4DIP-mediated tethering of PLCγ and PKCε on Golgi leads to constitutive activation of PKCε, and the sufficiently phosphorylated PKCε then dissociates from the Golgi and is released into the cytosol to promote NF1 degradation.

Approaches to target KRAS-mutant cancers with inhibitors of MEK have failed, often due to the induction of RTK genes and/or their ligands [38]. In this study, we identified PDE4DIP as a new driver of adaptive MEKi resistance in KRAS-mutant CRC. Our data demonstrated that MEKi treatment induced sustained upregulation of PDE4DIP expression. Furthermore, we found that knockdown of PDE4DIP restored the susceptibility of tumor cells to MEKi treatment. PDE4DIP knockdown also blocked the sustained reduction in the NF1 level and simultaneously impeded RAS overactivation and ERK reactivation in response to chronic MEK inhibition. Our study shows that PDE4DIP confers adaptive MEKi resistance and affects tumor growth through a similar NF1/RAS/ERK signaling axis. Intriguingly, we found that MEKi also induced sustained PLCγ activation and that knockdown of PDE4DIP impeded PLCγ overactivation. Conversely, blocking PLCγ activation attenuated MEKi-induced PDE4DIP overexpression. Our study suggests that a positive feedback loop between PDE4DIP and PLCγ is formed in MEKi-resistant cells. Taken together, our findings support the idea that a feedback mechanism involving PDE4DIP and PLCγ is initiated upon MEK inhibition; this feedback subsequently overactivates RAS signaling through suppression of NF1 sufficiently that even a MEKi cannot completely block the oncogenic RAS signaling leading to ERK reactivation. Thus, tumor cell proliferation is maintained. Our findings suggest that inhibition of PDE4DIP is a promising approach for overcoming MEKi resistance and improving the clinical outcome of CRC.

In summary, we report that PDE4DIP plays an oncogenic role in CRC tumorigenesis and MEKi resistance through modulation of the core RAS signaling pathway. Our study reveals a novel PDE4DIP-mediated PKCε/NF1/RAS signal transduction axis and highlights PDE4DIP as a promising therapeutic target for KRAS-mutant cancers.

## Materials and methods

### Tissue samples and cell lines

Fresh primary CRC tumors and matched adjacent normal mucosa were obtained from the First Affiliated Hospital of Wenzhou Medical University with appropriate Institutional Review Board approval and informed consent from patients. DLD1, SW480 and other CRC cell lines were originally obtained from the American Type Culture Collection (ATCC; USA). Routine cell cultures were maintained in RPMI-1640 medium supplemented with 10% fetal bovine serum (FBS) and 1% penicillin/streptomycin and incubated in a 5% CO_2_ atmosphere at 37 °C. SW480 cells were cultured in Leibovitz’s L-15 medium without CO_2_. The DLD1 and SW480 cell lines had recently been authenticated using short tandem repeat DNA profiling, and all cell lines tested negative for mycoplasma contamination before use in experiments.

### Reagents and antibodies

The PLCγ inhibitor U73122 (Cat# S8011), PKCε inhibitor EV1-2 (Cat# P1160), MEKi AZD6244 (Cat# S1008) and MEKi trametinib (Cat# S2673) were purchased from Selleck (Shanghai, China). siRNAs targeting the open reading frames of PDE4DIP, NF1, and AKAP450 were synthesized by GenePharma (Shanghai, China). The lentivirus containing a shRNA targeting PDE4DIP was designed and produced by GeneChem Co. (Shanghai, China). The siRNA and shRNA sequences are listed in Table S2. siRNA transfection was performed using Lipofectamine RNAiMAX (Invitrogen, Cat# 13778075) according to the manufacturer’s instructions, and cells were replated for further experiments. The knockdown efficiency was determined using qRT‒PCR or Western blotting. Antibodies against phospho-ERK (#9101), ERK (#9102), β-actin (#4970), phospho-AKT (#9271), AKT (#4691), phospho-P38 (#9211), phospho-JNK (#9255), JNK (#9252), phospho-MEK (#86128), MEK (#4694), GAPDH (#2118), Tubulin (#2144), PTEN (#9559), phospho-EGFR (#3777), EGFR (#2232), phospho-FAK (#8556), FAK (#71433), phospho-SRC (#59548), SRC (#2109), Myc tag-rabbit (#2278), Myc tag-mouse (#2276), PCNA (#13110), phospho-MARCKS (#11992), MARCKS (#5607), phospho-PKC (pan) (#2060), phospho-PLCγ (#2821), phospho-PKA (#4781), PKA (#4782), phospho-CREB (#9198), CREB (#9197), GM130 (#12480), IgG-rabbit (#2729), and IgG-mouse (#5415) were purchased from Cell Signaling Technology (Danvers, USA). Antibodies against P38 (sc-7972), NF1 (sc-67), the HA probe (sc-7392), phospho-PKCε (sc-12355), PKCε (sc-1681), PLC γ (sc-7290), p120GAP (sc-63), and PDE4D (sc-25814) were purchased from Santa Cruz Biotechnology (Santa Cruz, USA). The AKAP9 (611518) antibody was purchased from BD Transduction Laboratories (San Diego, USA). The PDE4DIP antibody (A89686) was purchased from Sigma‒Aldrich (St. Louis, USA). Detailed dilution and application information is provided in Table S3.

### Constructs and transfection

The Myc-tagged full-length PDE4DIP expression plasmid was purchased from OriGene Biology Company (Cat# RC214012). The coding sequence of NF1-GRD was amplified from HEK293 cells using a One-Step RT‒PCR System Kit (Invitrogen, Cat# 12574-018) and subcloned into the pcDNA3.1 vector. The coding sequence of PDE4DIP isoform 5 was amplified from DLD1 cells, sequenced and subcloned into the pcDNA3.1 vector. The primer sequences used for plasmid construction are listed in Table S2. Plasmid transfection was performed using Lipofectamine 2000 reagent (Invitrogen, Cat# 11668-019) according to the manufacturer’s instructions. To obtain stable PDE4DIP transfectants, cells transfected with the PDE4DIP expression plasmid were cultured in medium containing 1000 μg/ml G418, resistant clones were collected, and the expression of PDE4DIP was confirmed by Western blotting.

### Cell proliferation assays

Cell viability was determined with an MTT (Sigma) assay as described previously [50]. Cell proliferation was monitored in real time by using an RTCA-MP system (Acea Biosciences). CRC cells were seeded in an E-plate 16 at a density of 5000 cells per well and locked in an RTCA-MP device at 37 °C with 5% CO_2_. The cell index was monitored in real time every 15 min for 90 h, and the recorded curve shows the cell index ± SD values. For the colony formation assay, cells were seeded into 6-well plates at a density of 1 × 10^3^ cells per well and cultured in medium for 2 weeks. To evaluate drug cytotoxicity, cells were seeded into 6-well plates at a density of 1 × 10^4^ cells per well and cultured in medium containing the indicated drugs for 2 weeks. At the end of the experiment, the surviving colonies were fixed and stained with 0.5% crystal violet, and colonies with more than 50 cells were counted.

### Animal experiments

Animal studies were performed according to a protocol approved by the Institutional Animal Care and Use Committee of Wenzhou Medical University (No. wydw2019-0842). Five-week-old male athymic nude mice were purchased from Vital River Experimental Animal Center (Beijing, China), randomly divided into six groups (6 in each group) and housed under pathogen-free conditions. DLD1 or SW480 cells (3 × 10^6^ cells suspended in 200 μl of PBS) infected with lentivirus containing control shRNA (shNC) or an shRNA targeting PDE4DIP (shP1 or shP2) were injected subcutaneously into the dorsal flanks of mice. Tumors were measured every four days with a digital caliper, and tumor volumes were calculated using the standard equation, as follows: V = A × B^2^ × 0.5326 (A = long axis and B = short axis). At the end of the study, all mice were sacrificed, and the tumors were resected and weighed.

### Western blotting

Western blotting was performed as described previously [50]. In brief, cells were washed with PBS and lysed in RIPA buffer supplemented with complete protease inhibitors (Sigma, Cat# P8340) and phosphatase inhibitor cocktails (Roche, Cat# 04906845001). Protein quantification was performed with a BCA Protein Assay Kit (Pierce, Cat# 23225). Nuclear proteins were isolated using NE-PER™ Nuclear and Cytoplasmic Extraction Reagents (Pierce, Cat# 78833) according to the manufacturer’s instructions. Proteins were separated using sodium dodecyl sulfate‒polyacrylamide gel electrophoresis (SDS‒PAGE) and transferred onto polyvinylidene difluoride membranes (Bio-Rad, Cat# 1620177). After blocking with 5% milk in TBS containing 0.1% Tween 20 (TBST), the membranes were incubated first with the corresponding primary antibodies (1:1000~2000; specific dilutions are listed in Table S3) and then with horseradish peroxidase (HRP)-conjugated secondary antibodies. Protein bands were visualized with an Immun-Star HRP Chemiluminescence Kit (Bio-Rad, Cat# 1705061). Uncropped western bloting results were supplied in supplementary information.

### Coimmunoprecipitation

For endogenous immunoprecipitation, fresh cell pellets were lysed in cold cell lysis buffer (20 mM Tris-HCl, pH 7.5; 150 mM NaCl; 1.0% Triton X-100) containing freshly added protease inhibitor and incubated on ice for 20 min with vortexing. Then, the cell lysates were centrifuged at 12,000 rpm for 20 min to remove cell debris. Subsequently, the lysates were incubated first with the corresponding primary antibody overnight at 4 °C and then with 40 μl of Protein G-Sepharose (GE Healthcare, Cat# 17-0618-01) for 3 h at 4 °C. IgG was used as a negative control. After incubation, the beads were collected, washed four times with immunoprecipitation assay buffer, resuspended in Laemmli buffer and boiled for 5 min to release the bound proteins. Proteins in the eluted lysates were separated by SDS‒PAGE and were then analyzed by Western blotting as described above.

### Immunofluorescence assay

An immunofluorescence assay was performed to determine the localization and interaction of target proteins. Cells seeded on glass coverslips were fixed with 4% formaldehyde for 15 min, washed three times with PBS, and permeabilized with PBS containing 0.1% Triton X-100 for 10 min. The cells were subsequently blocked with 2% bovine serum albumin in PBS for 20 min. Next, the cells were incubated with anti-Myc (1:200), anti-PKCε (1:50), anti-PLCγ (1:50), anti-GM130 (1:1000), anti-PDE4D (1:100) and anti-NF1 (1:50) antibodies at 4 °C overnight. The next day, the coverslips were rinsed in PBS three times and were incubated with Alexa Fluor 488- or 594-conjugated secondary antibodies (diluted 1:500; Invitrogen, Cat# A11008 and A11005) for 2 h at room temperature. After three rinses in PBS, the coverslips were mounted on glass slides in the presence of 4′,6-diamidino-2-phenylindole (DAPI; Beyotime, Cat# C1002) for nuclear staining and were then imaged with a laser scanning confocal microscope.

### RAS GST-RBD pulldown assay

The abundance of the active GTP-bound form of RAS was analyzed using an Active RAS Pull-Down and Detection Kit (Thermo Fisher Scientific, Cat# 16117) according to the manufacturer’s instructions. Cell lysates containing 1.0 mg of protein were incubated with 50 μl of Raf1-RBD-agarose beads to pull down activated GTP-bound RAS. Then, the beads were pelleted, washed three times with 1× Assay Buffer, and resuspended in 50 μl of 2× reducing SDS‒PAGE sample buffer. Subsequently, after denaturation by boiling and centrifugation, supernatant samples were analyzed by Western blotting using the supplied KRAS-specific polyclonal antibody. In addition, whole-cell lysates (10 µg) were subjected to Western blot analysis for evaluation of total KRAS.

### IHC staining

CRC biopsy samples were paraffin-embedded and sectioned at 4 μm for staining and histologic analysis. For staining, slides containing paraffinized tissue sections were deparaffinized and rehydrated, and antigen retrieval was performed in citrate buffer (10 mmol/L sodium citrate, 0.05% Tween 20; pH 6.0). The sections were treated with 3% H_2_O_2_ and incubated with blocking buffer for 1 h. The tissue sections were then incubated with a primary antibody against PDE4DIP (1:200) followed by a biotinylated, peroxidase-conjugated secondary antibody. The stained slides were visualized and imaged with a Zeiss microscope with a 40× objective lens.

### Cycloheximide chase assay

To examine the effect of PDE4DIP on NF1 stability, DLD1 cells were treated with cycloheximide at a final concentration of 100 μg/ml. The reaction was terminated at the 0-, 1-, 2- and 3-h time points as indicated. The protein concentrations were normalized, and 20 μg of total protein from each sample was immunoblotted with different antibodies. Densitometric analysis of the resulting bands was performed by using ImageJ software (http://rsb.info.nih.gov/ij), and protein abundances were normalized to that of α-tubulin. The final values presented are the averages of the results from three independent densitometric analyses.

### Ubiquitination assay

Cells were transfected with the HA-Ubiquitin plasmid. Twenty-four hours after transfection, the cells were treated with the proteasome inhibitor MG132 (25 mM) (Sigma, Cat# M7449) for 4 h and were then lysed in ubiquitination assay buffer containing protease/phosphatase inhibitors. Cell lysates were clarified and incubated with an anti-NF1 antibody overnight at 4 °C. Immunocomplexes were incubated with Protein G-Sepharose (GE Healthcare, Cat# 17-0618-01) for another 3 h at 4 °C, washed four times with wash buffer, and boiled for 5 min in Laemmli buffer before separation by SDS‒PAGE. Western blotting was performed with an anti-HA antibody to detect ubiquitinated NF1.

### Real-time PCR (qRT‒PCR) analysis

Total RNA from cells or tissue samples was isolated with TRIzol Reagent (Invitrogen, Cat# 15596018), and cDNA synthesis was performed using an M-MLV reverse transcriptase kit (Invitrogen, Cat# 28025-013) according to the manufacturer’s instructions. qRT‒PCR was carried out with SYBR Green (Tiangen, China, Cat# FP202-02#) in biological triplicate in an ABI 7500 Real-Time detection system (Applied Biosystems). After the reactions were complete, relative gene expression levels were calculated using the 2^−ΔΔCt^ method. GAPDH was used as an endogenous control. The primer sequences used to amplify the mRNAs are listed in Table S2.

### RNA stability assay

To measure the stability of NF1 mRNA, 5 g/mL of actinomycin D (MedChemExpress, USA, Cat#HY-17559) was added to the cell culture to inhibit transcription. RNA of DLD1 and SW480 cells was harvested after incubation for 0 h, 5 h, 10 h, and 15 h, and was subjected to qRT-PCR analysis for NF1 expression at mRNA level.

### RNA sequencing (RNA-seq) and data analysis

For RNA-seq, purified RNA from shPDE4DIP and control cells was used for library construction with an Illumina TruSeq RNA Sample Prep Kit (FC-122-1001). The libraries were then sequenced on the Illumina HiSeq 4000 or X Ten platform (BGI-Shenzhen, China). The raw reads were aligned to the human genome GRCh37/hg19 with Bowtie2. Differential gene expression was identified using the PossionDis method, which was performed as described: FC (fold change) ≥ 2.00 and FDR ≤ 0.001. Phyper (https://en.wikipedia.org/wiki/Hypergeometric_distribution) was used to perform KEGG and GO enrichment analyses. Differentially expressed genes are listed in Table S4. The RNA-seq data are available under NCBI Bioproject PRJNA895757 and BioSample SAMN31523744 (SW480_shNC) and SAMN31523746 (SW480_shPDE4DIP).

### Statistical analysis

All data are presented as the means ± SDs unless otherwise stated. GraphPad Prism 8.0 and SPSS Statistics software (SPSS 20) were used to perform all statistical analyses. The significance of differences between groups was evaluated by Student’s t test or ANOVA. Clinical association analysis was performed by the chi-square test. P < 0.05 was considered statistically significant.

## Supplementary information

Supplemental figure S1-8 and table S1-3

Table S4.Differential gene expression in SW480 shPDE4DIP and control cells

Original Data File

aj-checklist

## Data Availability

The RNA-seq data are available under NCBI Bioproject PRJNA895757 and BioSample SAMN31523744 (SW480_shNC) and SAMN31523746 (SW480_shPDE4DIP). The datasets generated and/or analysed during the current study are available from the corresponding authors on reasonable request.
