# Supplementary material for: PDE4DIP contributes to colorectal cancer growth and chemoresistance through modulation of the NF1/RAS signaling axis
Source: Cell Death Dis. 2023 Jun 24;14(6):373. doi: 10.1038/s41419-023-05885-y (PMC10290635; doi:10.1038/s41419-023-05885-y)
Supplement: Supplementary file 1 — Supplemental figure S1-8 and table S1-3 [file 41419_2023_5885_MOESM1_ESM.pdf]

Supplemental Materials

Title: **PDE4DIP contributes to colorectal cancer growth and chemoresistance through modulation of the NF1/RAS signaling axis**

Pan R. et al.

Supplemental figure legends

Figure S1. Expressions of PDE4DIP and its variants in CRC cells.

(A) Western blot analysis of the expression of PDE4DIP variants in nine CRC cell lines. The full-length PDE4DIP (PI) and isoform-5 (PI-5) were commonly expressed in CRC cells. (B) shRNA-mediated knockdown of PDE4DIP expression (PI and PI-5) was examined by Western blot. DLD1 or SW480 cells were infected with lentivirus containing control (shNC) or shRNAs targeting PDE4DIP (shP1, shP2) for 72 h. (C) MTT assay showing knockdown of PDE4DIP isoform-5 have no effect on CRC cell proliferation. Top, siRNA-mediated specifically silencing of PDE4DIP isoform-5 (PI-5) was validated by Western blot, DLD1 or SW480 cells were transfected with negative control siRNA (siNC) or two siRNA targeting PDE4DIP isoform-5 (siPI5-1#, siPI5-2#) for 48 h.; Bottom, data represent mean ± SD from three biological replicates. (D) MTT assay showing overexpression of PDE4DIP isoform-5 has no effect on CRC cell proliferation. Top, ectopic overexpression of PDE4DIP isoform-5 (PI-5) was validated by Western blot; Bottom, data represent mean ± SD from three biological replicates.

Figure S2. Effects of PDE4DIP knockdown on PDE4D/PKA and other signaling pathways in CRC cells.

(A) Western blot analysis of the activity of PDE4D/PKA signaling in PDE4DIP-silenced CRC cells. Cells were transfected with negative control siRNA (siNC) and two siRNA targeting PDE4DIP (siP1, siP2) for 48 h. (B) Immunofluorescence showing the colocalization of PDE4DIP (red) and GM130 (green) at Golgi apparatus. DLD1 or SW480 cells were transfected with PDE4DIP expressing plasmid for 48 h. Scale bars, 5 μm. (C) Subcellular localization of PDE4DIP (red) and PDE4D (green). DLD1 or SW480 cells were transfected with PDE4DIP expressing plasmid for 48 h. Scale bars, 5 μm. (D) Gene Ontology (GO) analysis of the pathways enriched in PDE4DIP-silenced SW480 cells.

Figure S3. GSEA analysis of the correlation between PDE4DIP expression and MAPK/AKT signaling in CRC.

(A) GSEA plots depicting enrichment in MAPK or AKT/mTOR pathways based on PDE4DIP expression in CRC tumor samples from TCGA datasheet. False discovery rate (FDR) was set at 0.25 and significance was considered as $p < 0.05$. (B-C) GSEA analysis of enrichment in MAPK and AKT/mTOR pathway based on PDE4DIP expression in CRC tumors derived from GSE39582 cohort and GSE14333 cohort. FDR was set at 0.25 and significance was defined as $P < 0.05$.

Figure S4. PDE4DIP promotes CRC growth through RAS-ERK/AKT pathway.

(A) MTT assay showing inhibition of ERK or AKT attenuated the PDE4DIP-potented CRC cell proliferation. DLD1 and SW480 cells transfected with empty vector (Vec) or

Myc-tagged PDE4DIP expressing plasmid (PI) were treated with or without LY294002 (20 μM) or U0126 (2 0 μM) fo r 24 h. Representative of at least three independent experiments performed in triplicate. Data are mean ± SD, and *P* values were determined by Student's *t* tests. *P* ** < 0.01, *P* *** < 0.001. (B) Western blot showing inhibition of RAS suppressed ERK/AKT phosphorylation in DLD1 and SW480 cells. Cells were treated with DMSO or FTS (salirasib, 75 μm) for 72 h. (C) Kaplan-Meier curves of overall survival in CRC patients according to PDE4DIP expressions and KRAS mutation status. CRC patients from the GSE40967 cohort were stratified into two groups based on KRAS mutation status: KRAS-mutated (left) or KRAS-wild-type (right). Survival significance was tested by log-rank Mantel-Cox test.

Figure S5. NF1 suppresses oncogenic RAS/ERK signaling and KRAS-mutant CRC cell growth.

(A) Western blot analysis of the levels of phosphorylated FAK (pFAK), FAK, phosphorylated SRC (pSRC), SRC, and p120GAP in control (siNC) and PDE4DIP-silenced (siP1, siP2) CRC cells. (B) Western blot showing silencing of NF1 enhanced RAS activity, ERK and AKT phosphorylation in KRAS-mutant DLD1 and SW480 cells. Cells were transfected with negative siRNA (siNC) or siRNA targeting NF1 (siNF1). (C) Western blot showing ectopic expression of NF1 suppressed RAS activity and ERK phosphorylation in DLD1 and SW480 cells. Cells were transfected with empty vector (Vec) or NF1-GRD overexpression plasmid for 48 h. (D) MTT assay showing ectopic expression of NF1 suppressed DLD1 and SW480 cell

proliferation. Cells were transfected with empty vector (Vec) or the indicated concentrations of NF1-GRD plasmid. (E) Colony formation assay showing pre-silencing of NF1 (siNF1) attenuated the inhibitory effect of PDE4DIP interference on cell proliferation. (D-E), Representative of at least three independent experiments performed in triplicate. (F) RNA stability assay showing knockdown of PDE4DIP had no effect on the mRNA stability of NF1 in DLD1 and SW480 cells. Data are mean ± SD, and *P* values were determined by Student's *t* tests. *P* ** < 0.01, *P* *** < 0.001.

Figure S6. PKCε promotes the degradation of NF1 in CRC cells.

(A) Western blot analysis of phosphorylation of MARCKS (pMARCKS) in control and PDE4DIP-overexpressing CRC cells. DLD1 or SW480 cells were transfected with empty vector (Vec) or Myc-tagged PDE4DIP expressing plasmid (PI) for 48 h. (B) Western blot showing inhibition of PKC increase the NF1 levels in CRC cells. DLD1 and SW480 cells were treated with RO-31-8220 (0, 5, 10 μM) for 24 h. (C) Western blot showing inhibition of PKCε increase the expression of NF1 in CRC cells. DLD1 and SW480 cells were treated with PKCε inhibitor EV1-2 (1.0 μM) for the indicated time points. (D) Western blot showing inhibition of PKCε dose-dependently affects NF1 expression and ERK phosphorylation in DLD1 and SW480 cells. Cells were stimulated with indicated concentrations of EV1-2 for 30 min. (E) Immunofluorescence showing the abundance of NF1 in control and EV1-2 treated CRC cells. Cells were stimulated with DMSO or 1.0 μM EV1-2 for 30 min. Scale bars, 20 μm. (F) Western blot analysis of NF1 ubiquitination in control (DMSO) and EV1-2

treated CRC cells (1.0 μM, 30min). (G) MTT assay showing knockdown of PKCε suppressed the proliferation of DLD1 and SW480 cells. Cells were transfected with negative control siRNA (siNC) or two siRNA targeting PKCε (si-ε1, si-ε2). Top, siRNA-mediated knockdown of PKCε expression was validated by Western blot; Bottom, data represent mean ± SD from three biological replicates. *P* values were determined by Student's *t* tests. *P* ** < 0.01, *P* *** < 0.001.

Figure S7. PDE4DIP interacts with AKAP9 at Golgi apparatus and promotes the activation of PLCγ.

(A) Coimmunoprecipitation of PDE4DIP with AKAP9 in DLD1 and SW480 cells. Cells were transfected with Myc-tagged PDE4DIP expressing plasmid, and cell lysates were immunoprecipitated with an anti-Myc antibody and probed with anti-Myc and anti-AKAP9 antibodies. (B) Western blot showing the activation of PLCγ in PDE4DIP-overexpressing CRC cells. DLD1 or SW480 cells were transfected with empty vector (Vec) or PDE4DIP expressing plasmid (PI) for 48 h.

Figure S8. Inhibition of PLCγ activation sensitizes KRAS-mutant CRC cells to MEK inhibition.

(A) Dose-response curves of control and PDE4DIP-silenced DLD1 and SW480 cells treated with MEK inhibitor Trametinib. Cells infected with lentivirus containing control (shNC) or shRNAs targeting PDE4DIP (shP1, shP2) were treated with increasing concentrations of Trametinib for 72 h. Representative of at least three

independent experiments performed in triplicate. Data are mean ± SD, and *P* values were determined by Student's *t* tests. *P* ** < .001, *P* *** < 0.001 vs. control. (B) Colony formation assay of control and PDE4DIP-silenced DLD1 and SW480 cells treated with indicated concentrations of Trametinib for two weeks. Images are representative of at least three independent experiments. (C) Western blot analysis of NF1 level and activation of PLCγ and ERK in AZD6244-resistant CRC cells treated with AZD6244 (1.0 μM) in combination with or without a PLCγ inhibitor (U73122, 1.0 μM). Cells were collected for lysis at the indicated time points. UT, untreated. (D) Colony formation assay of DLD1 and SW480 cells treated with AZD6244, a PLCγ inhibitor (U73122, 1.0 μM), or a combination. Images are representative of at least three independent experiments.

Table S1. Correlation between alterations of PDE4DIP expression in CRC tumors and clinical and pathologic features of the individuals.

Clinical characteristic	Patient No.	PDE4DIP mRNA upregulation		χ^2	*P* value
		Yes (48)	No (12)		
**Sex**					
Male	32	27	5	0.82	0.365
Female	28	21	7		
Age (*y*)					
<60	24	22	2	2.296	0.13
≥60	36	26	10		
**Differentiation grade**					
Well	13	8	5	4.284	0.117
Moderate	33	27	6		
Poor	14	13	1		
**Infiltration depth**					
T1	7	1	6	21.716	<0.001
T2	28	24	4		
T3	12	11	1		
T4	13	12	1		
**Lymph node metastasis**					
N0	23	14	9	8.674	0.013
N1	19	17	2		
N2	18	17	1		
**TNM stage**					
I	19	12	7	8.8889	0.012
II	4	2	2		
III	37	34	3		
**Tumor size (cm)**					
<5	42	31	11	2.188	0.139
≥5	18	17	1		
**Smoking**					
Yes	22	18	4	0	1
No	38	30	8		
**Drinking**					
Yes	25	21	4	0.429	0.513
No	35	27	8		

Note: Using SPSS 20 statistical software, a chi-squared analysis was performed to analyze the correlations between PDE4DIP mRNA levels and clinical and pathologic parameters. The

statistical significance of the individuals was determined with chi-squared test (χ^2). $P < 0.05$ was considered significant. Tumor size, differentiation grade, infiltration depth and lymph node metastasis were determined by the pathologists. Tumor stage was based on TNM.

Table S2. Sequences used for siRNA, shRNA interference, qRT-PCR and cloning.

Gene	Sequence (5'-3')	Application
PDE4DIP (siP1)	AGAGCGAGAUCAUGACUUATT	Gene silence
PDE4DIP (siP2)	AAGCAGAGAGACAGCUCUAUA	Gene silence
PDE4DIP (shP1)	AGAGCGAGAUCAUGACUUATT	Gene silence
PDE4DIP (shP2)	AAGCAGAGAGACAGCUCUAUA	Gene silence
PDE4DIP isoform 5 (siPI5-1#)	GGCUUCCGAUUCCAGUGAA	Gene silence
PDE4DIP isoform 5 (siPI5-2#)	GCCAGUAUGUAUCGGAAGATT	Gene silence
NF1 (siNF1)	CUUCGGAAUUCUGCCUCUG	Gene silence
AKAP450 (siA1/siAKAP9)	AACUUUGAAGUUAACUAUCAA	Gene silence
AKAP450 (siA2)	GCACAAUAAUUAUUGAAUUTT	Gene silence
PKCε (si-ε1)	UUGUAUAACCCAUGUUUAGCCTT	Gene silence
PKCε (si-ε2)	AGUGUAUACAGCUAAUUGCUGTT	Gene silence
Nontarget control siRNA (siNC)	UUCUCCGAACGUGUCACGUTT	Gene silence
Nontarget control shRNA (shNC)	UUCUCCGAACGUGUCACGUTT	Gene silence

PDE4DIP	Forward:<br>5’-GAGAACTCCAGGACAAGAAACAGCAT-3’<br>Reverse:<br>5’-GGATTCCTCCTGCAGAAGCTGG-3’	qRT-PCR
NF1	Forward:<br>5’-CAGAATTCCCCCCTCAACTTCGAAGT-3’<br>Reverse:<br>5’-TGCGTGCTGCATCAAAGTTGCTTTTCAC-3’	qRT-PCR
NF1-GRD	Forward:<br>5’-CCAAGCTTGCCACCATGGGTTACCACAAGGATCTCCAG-3’<br>Reverse:<br>5’-CGTCTAGAGTGCTCTGGAGGACCCAGGTA-3’	Cloning
PDE4DIP isoform 5 (PI5)	Forward:<br>5’-CGGATCCGCCACCATGAAGGAGATTTGCAGGATC-3’<br>Reverse:<br>5’-CCTCGAGTAGTTGCTGGTGACTATGGTGT-3’	Cloning

Table S3. Primary antibodies used.

Antibody	LOT	KD	Host	Company	Application
PDE4DIP	A89686	150-300	Rabbit	Sigma	WB 1:2000<br>IHC 1:200<br>IF 1:200
Phospho-p44/42 MAPK(Erk1/2)(Thr202/Tyr 204) Antibody	#9101	42,44	Rabbit	CST	WB 1:2000
p44/42 MAPK(Erk1/2) Antibody	#9102	42,44	Rabbit	CST	WB 1:2000
β-actin	#4970	45	Rabbit	CST	WB 1:2000
Phospho-Akt (Ser473) Antibody	#9271	60	Rabbit	CST	WB 1:2000
Akt(pan)(C67E7) Rabbit mAb	#4691	60	Rabbit	CST	WB 1:2000
Phospho-p38 MAPK (Thr180/Tyr182) Antibody	#9211	38	Rabbit	CST	WB 1:2000
P38 alpha/beta MAPK(A-12)	sc-7972	38	Mouse	Santa	WB 1:2000
Phospho-SAPK/JNK(Thr183 /Tyr185)(G9) Mouse mAb	#9255	46,54	Mouse	CST	WB 1:2000
SAPK/JNK Antibody	#9252	46,54	Rabbit	CST	WB 1:2000

Phospho-MEK1/2 (Ser217/221) (E4M5C) Rabbit mAb	#86128	45	Rabbit	CST	WB 1:2000
MEK1/2 (L38C12) Mouse mAb	#4694	45	Mouse	CST	WB 1:2000
GAPDH(14C10) Rabbit mAb	#2118	37	Rabbit	CST	WB 1:6000
α-Tubulin Antibody	#2144	52	Rabbit	CST	WB 1:6000
PTEN(138G6) Rabbit mAb	#9559	54	Rabbit	CST	WB 1:2000
p-EGFR(Tyr1068) (D7A5)	#3777	175	Rabbit	CST	WB 1:2000
EGFR	#2232	175	Rabbit	CST	WB 1:2000
Neurofibromin (D)	sc-67	250	Rabbit	Santa	WB 1:1000<br>IF 1:200
Myc-tag Rabbit mAb	#2278		Rabbit	CST	WB 1:2000<br>IF 1:200
Myc-tag Mouse mAb	#2276		Mouse	CST	WB 1:2000<br>IF 1:200
HA-probe(F-7)	sc-7392		Mouse	Santa	WB 1:1000
PCNA (D3H8P)<br>XP® Rabbit mAb	#13110	36	Rabbit	CST	WB 1:2000
Phospho-MARCKS (Ser159/163) (D13D2) Rabbit mAb	#11992	80	Rabbit	CST	WB 1:1000
MARCKS(D88D11)	#5607	75	Rabbit	CST	WB 1:2000
p-PKC-ε(Ser729)	sc-12355	90	Mouse	Santa	WB 1:1000
PKC ε(E-5)	sc-1681	90	Mouse	Santa	WB 1:1000<br>IF 1:200
Phospho-PKC (pan) (zeta Thr410) (190D10) Rabbit mAb	#2060	76,85	Rabbit	CST	WB 1:1000
AKAP9	611518	450	Mouse	BD transduction laboratories	WB 1:800<br>IP 1:100
Phospho-PLCγ1 (Tyr783) Antibody	#2821	155	Rabbit	CST	WB 1:1000
PLC γ1 (E-12)	sc-7290	155	Mouse	Santa	WB 1:1000<br>IF 1:200
PDE4D(H-69)	sc-25814	68	Rabbit	Santa	WB 1:2000
Phospho-PKA C (Thr197)	#4781	42	Rabbit	CST	WB 1:2000

Antibody					
PKA C-α Antibody	#4782	43	Rabbit	CST	WB 1:2000
Phospho-CREB (Ser133) (87G3) Rabbit mAb	#9198	43	Rabbit	CST	WB 1:2000
CREB (48H2) Rabbit mAb	#9197	43	Rabbit	CST	WB 1:2000
GM130 (D6B1) XP® Rabbit mAb	#12480		Rabbit	CST	IF 1:200
Normal Rabbit IgG	#2729		Rabbit	CST	IP 1:100
Mouse mAb IgG1 Isotype Control	#5415		Mouse	CST	IP 1:100
p120GAP (B4F8)	sc-63	120	Mouse	Santa	WB 1:2000
Phospho-FAK (Tyr397) Antibody	#8556	125	Rabbit	CST	WB 1:1000
FAK	#71433	125	Rabbit	CST	WB 1:2000
Phospho-SRC (Tyr416) Antibody	#59548	60	Rabbit	CST	WB 1:1000
SRC	#2109	60	Rabbit	CST	WB 1:2000

Figure S1

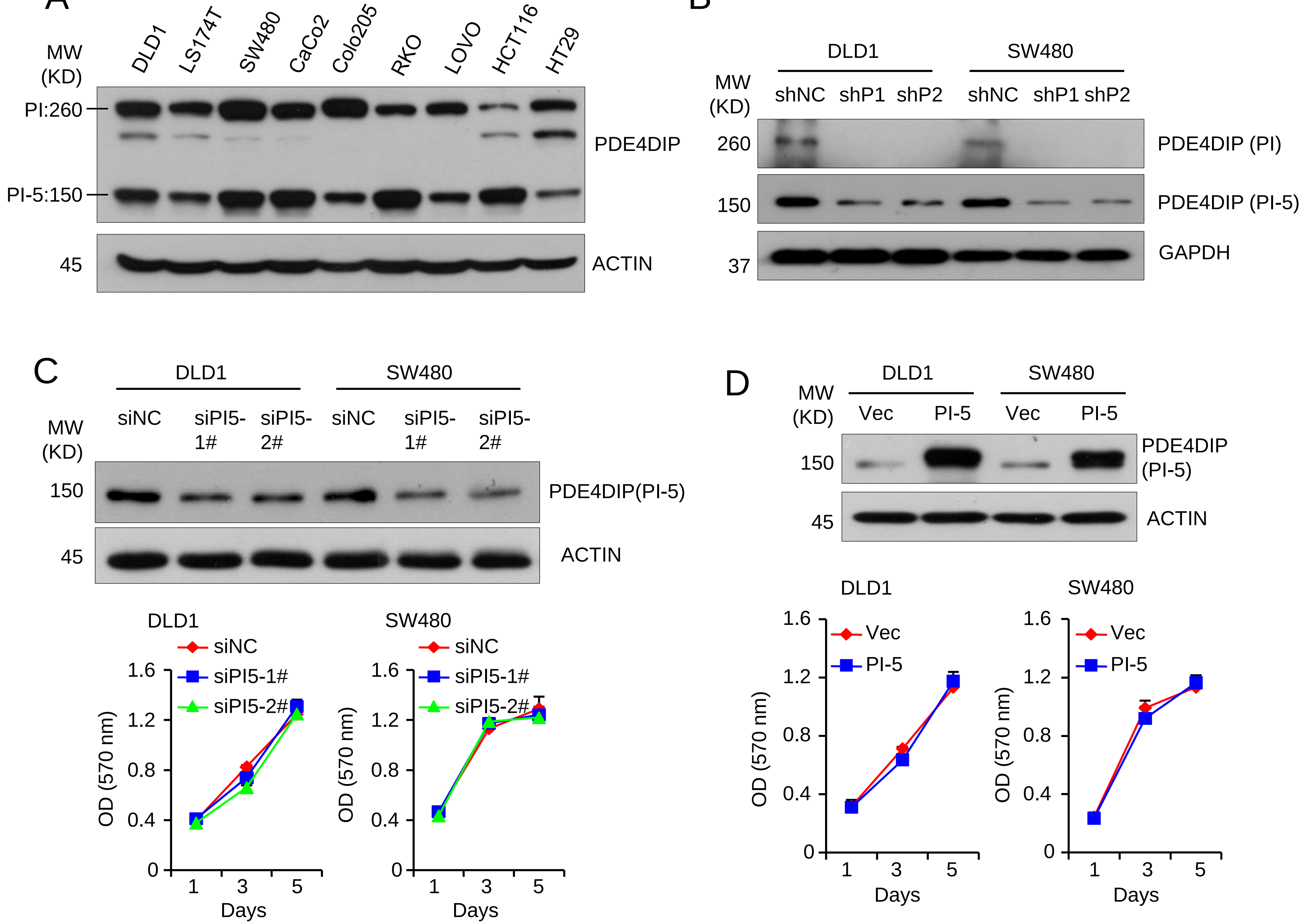

A
MW (KD)
DLD1
LS174T
SW480
CaCo2
Colo205
RKO
LOVO
HCT116
HT29
PI:260
PI-5:150
45
PDE4DIP
ACTIN
B
DLD1
SW480
MW (KD)
shNC
shP1
shP2
shNC
shP1
shP2
260
150
37
PDE4DIP (PI)
PDE4DIP (PI-5)
GAPDH
C
DLD1
SW480
MW (KD)
siNC
siPI5-1#
siPI5-2#
siNC
siPI5-1#
siPI5-2#
150
45
PDE4DIP(PI-5)
ACTIN
DLD1
SW480
siNC
siPI5-1#
siPI5-2#
OD (570 nm)
Days
D
DLD1
SW480
MW (KD)
Vec
PI-5
Vec
PI-5
150
45
PDE4DIP (PI-5)
ACTIN
DLD1
SW480
Vec
PI-5
OD (570 nm)
Days

Figure S2

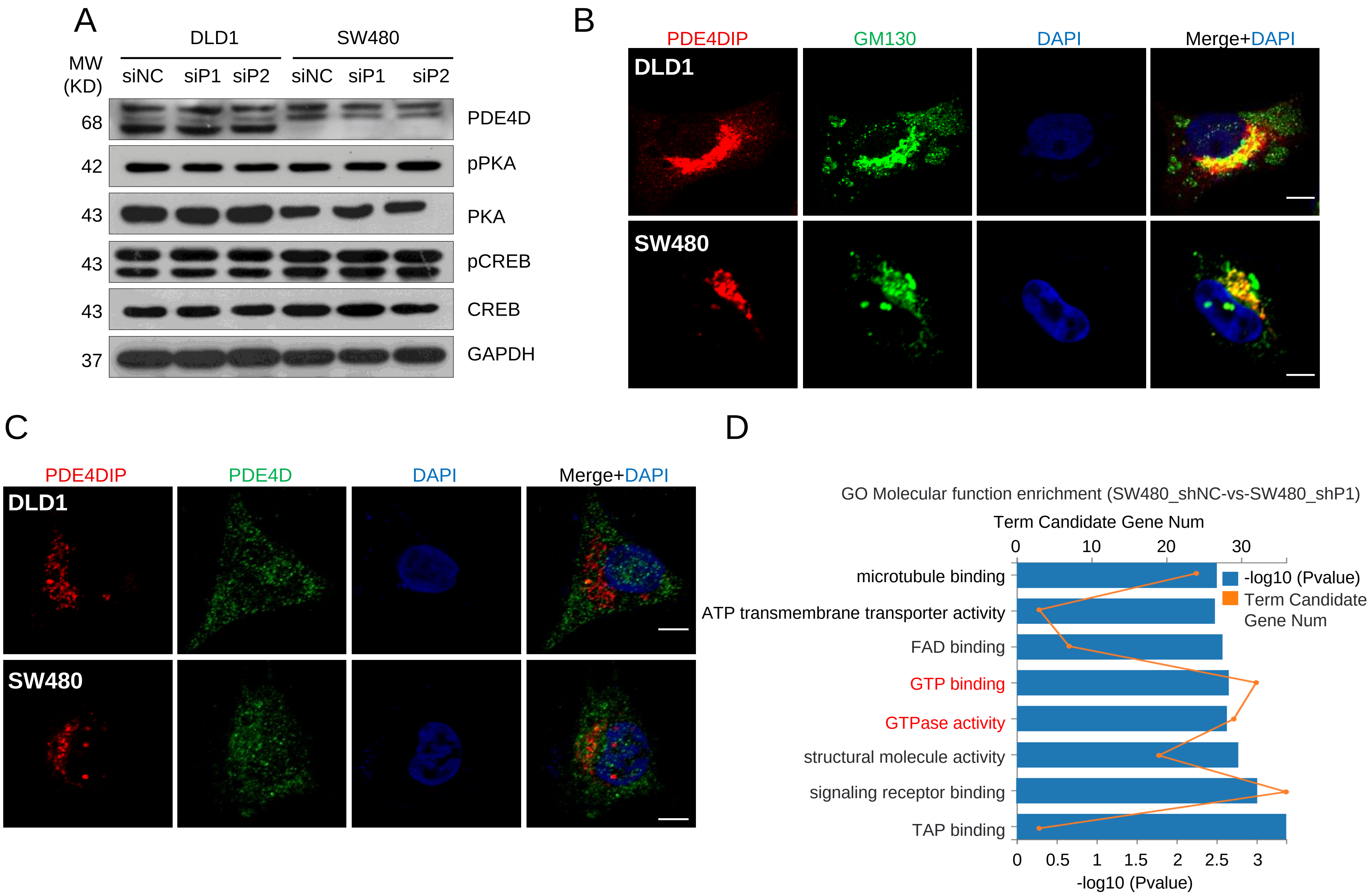
A
DLD1
SW480
MW (KD)
siNC
siP1
siP2
siNC
siP1
siP2
68
42
43
43
43
37
PDE4D
pPKA
PKA
pCREB
CREB
GAPDH
B
PDE4DIP
GM130
DAPI
Merge+DAPI
DLD1
SW480
C
PDE4DIP
PDE4D
DAPI
Merge+DAPI
DLD1
SW480
D
GO Molecular function enrichment (SW480_shNC-vs-SW480_shP1)
Term Candidate Gene Num
0
10
20
30
microtubule binding
ATP transmembrane transporter activity
FAD binding
GTP binding
GTPase activity
structural molecule activity
signaling receptor binding
TAP binding
-log10 (Pvalue)
Term Candidate Gene Num
0
0.5
1
1.5
2
2.5
3
-log10 (Pvalue)

Figure S3

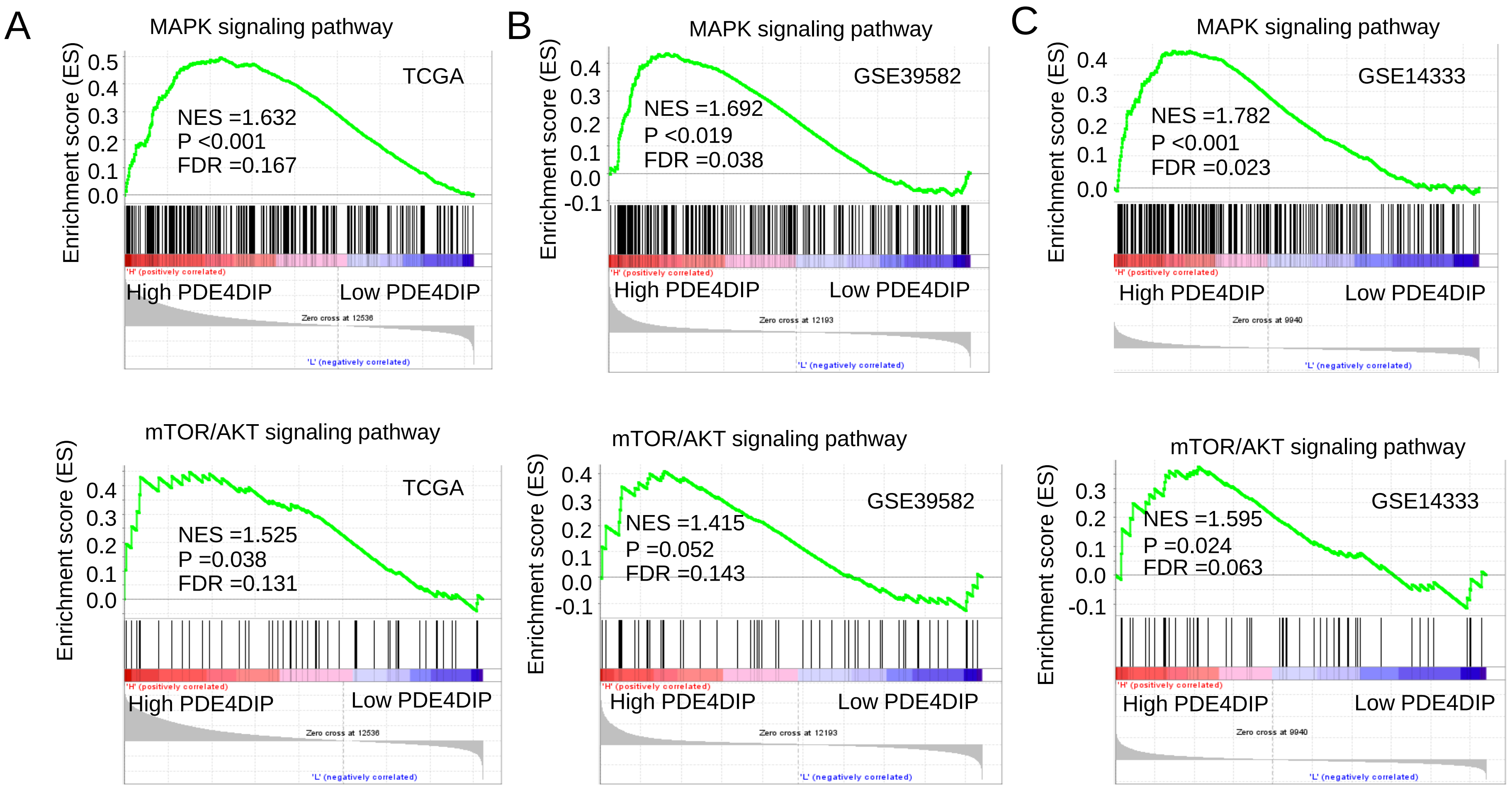
A
MAPK signaling pathway
TCGA
Enrichment score (ES)
NES =1.632
P <0.001
FDR =0.167
High PDE4DIP
Low PDE4DIP
Zero cross at 12536
B
MAPK signaling pathway
GSE39582
NES =1.692
P <0.019
FDR =0.038
High PDE4DIP
Low PDE4DIP
Zero cross at 12193
C
MAPK signaling pathway
GSE14333
NES =1.782
P <0.001
FDR =0.023
High PDE4DIP
Low PDE4DIP
Zero cross at 9940
mTOR/AKT signaling pathway
TCGA
NES =1.525
P =0.038
FDR =0.131
mTOR/AKT signaling pathway
GSE39582
NES =1.415
P =0.052
FDR =0.143
mTOR/AKT signaling pathway
GSE14333
NES =1.595
P =0.024
FDR =0.063
'H' (positively correlated)
'L' (negatively correlated)

Figure S4

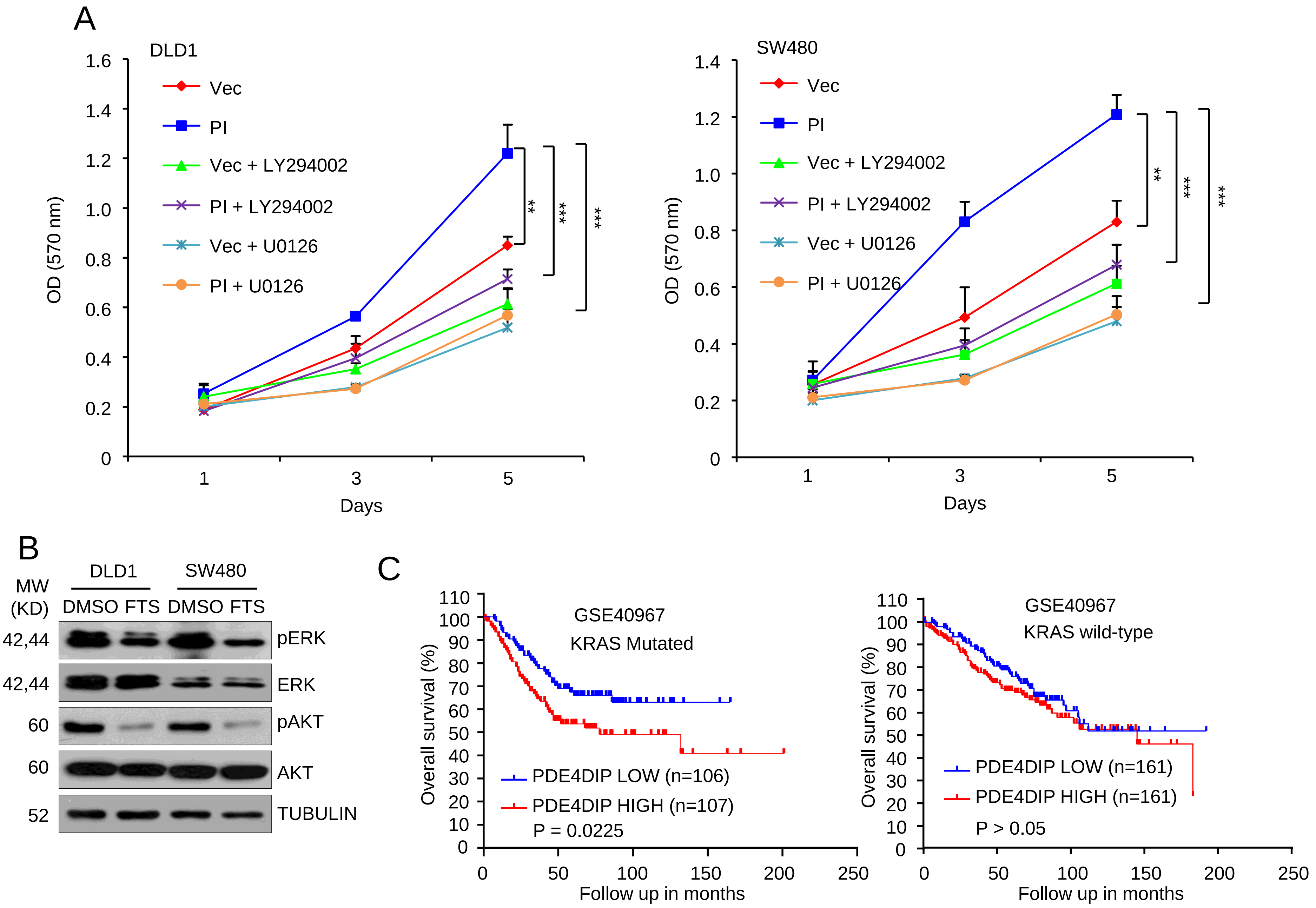
A
DLD1
1.6
1.4
1.2
1.0
0.8
0.6
0.4
0.2
0
OD (570 nm)
Vec
PI
Vec + LY294002
PI + LY294002
Vec + U0126
PI + U0126
**
***
***
1
3
5
Days
SW480
1.4
1.2
1.0
0.8
0.6
0.4
0.2
0
OD (570 nm)
Vec
PI
Vec + LY294002
PI + LY294002
Vec + U0126
PI + U0126
**
***
***
1
3
5
Days
B
MW (KD)
DLD1
SW480
DMSO FTS DMSO FTS
42,44 pERK
42,44 ERK
60 pAKT
60 AKT
52 TUBULIN
C
GSE40967
KRAS Mutated
Overall survival (%)
110
100
90
80
70
60
50
40
30
20
10
0
PDE4DIP LOW (n=106)
PDE4DIP HIGH (n=107)
P = 0.0225
0
50
100
150
200
250
Follow up in months
GSE40967
KRAS wild-type
Overall survival (%)
PDE4DIP LOW (n=161)
PDE4DIP HIGH (n=161)
P > 0.05
Follow up in months

Figure S5

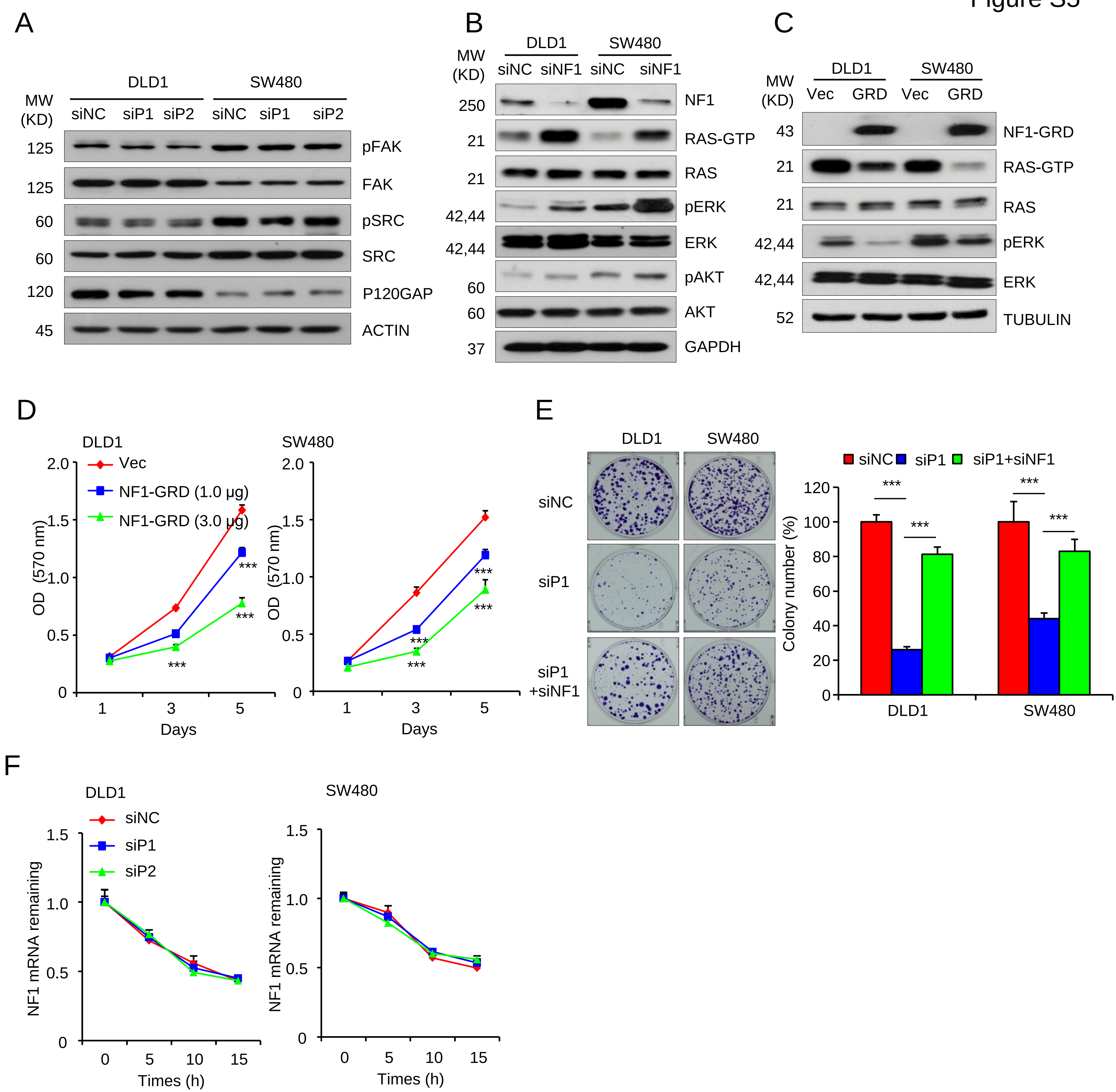
A
DLD1
SW480
MW (KD)
siNC siP1 siP2 siNC siP1 siP2
125 pFAK
125 FAK
60 pSRC
60 SRC
120 P120GAP
45 ACTIN
B
DLD1
SW480
MW (KD)
siNC siNF1 siNC siNF1
250 NF1
21 RAS-GTP
21 RAS
42,44 pERK
42,44 ERK
60 pAKT
60 AKT
37 GAPDH
C
DLD1
SW480
MW (KD)
Vec GRD Vec GRD
43 NF1-GRD
21 RAS-GTP
21 RAS
42,44 pERK
42,44 ERK
52 TUBULIN
D
DLD1
SW480
Vec
NF1-GRD (1.0 μg)
NF1-GRD (3.0 μg)
OD (570 nm)
Days
***
E
DLD1
SW480
siNC
siP1
siP1 +siNF1
siNC siP1 siP1+siNF1
Colony number (%)
***
F
DLD1
SW480
siNC
siP1
siP2
NF1 mRNA remaining
Times (h)

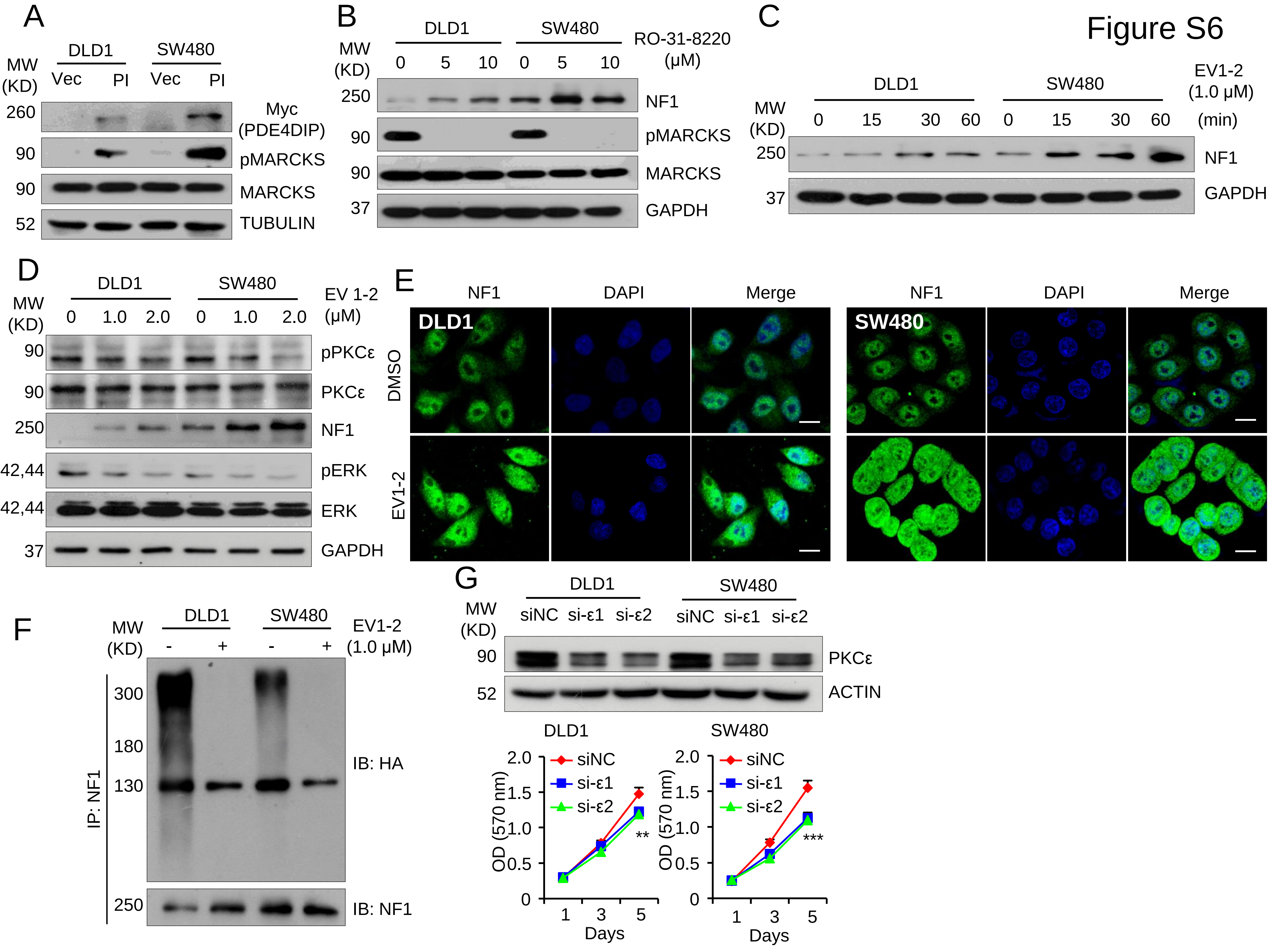
Figure S6
A
DLD1
SW480
MW (KD)
Vec
PI
Vec
PI
260
Myc (PDE4DIP)
90
pMARCKS
90
MARCKS
52
TUBULIN
B
DLD1
SW480
RO-31-8220 (μM)
MW (KD)
0 5 10 0 5 10
250
NF1
90
pMARCKS
90
MARCKS
37
GAPDH
C
DLD1
SW480
EV1-2 (1.0 μM)
MW (KD)
0 15 30 60 0 15 30 60
(min)
250
NF1
37
GAPDH
D
DLD1
SW480
EV 1-2 (μM)
MW (KD)
0 1.0 2.0 0 1.0 2.0
90
pPKCε
90
PKCε
250
NF1
42,44
pERK
42,44
ERK
37
GAPDH
E
NF1
DAPI
Merge
DLD1
SW480
DMSO
EV1-2
F
MW (KD)
DLD1
SW480
EV1-2 (1.0 μM)
- + - +
IP: NF1
300
180
130
IB: HA
250
IB: NF1
G
DLD1
SW480
MW (KD)
siNC si-ε1 si-ε2 siNC si-ε1 si-ε2
90
PKCε
52
ACTIN
DLD1
SW480
siNC
si-ε1
si-ε2
OD (570 nm)
2.0
1.5
1.0
0.5
0
1 3 5
Days
**
***

Figure S7

A

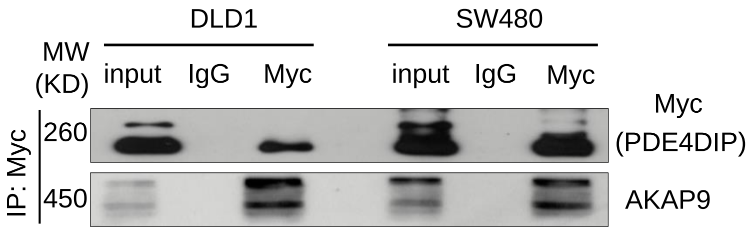
DLD1
SW480
MW
(KD)
input
IgG
Myc
input
IgG
Myc
IP: Myc
260
450
Myc
(PDE4DIP)
AKAP9

B

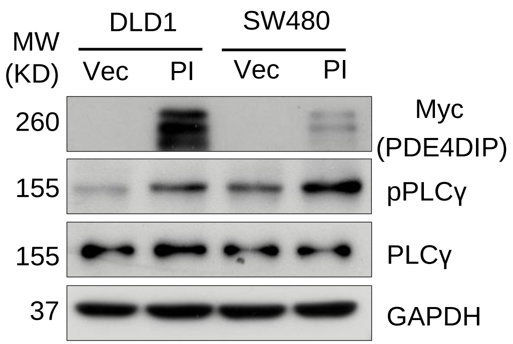
DLD1
SW480
MW
(KD)
Vec
PI
Vec
PI
260
155
155
37
Myc
(PDE4DIP)
pPLCγ
PLCγ
GAPDH

Figure S8

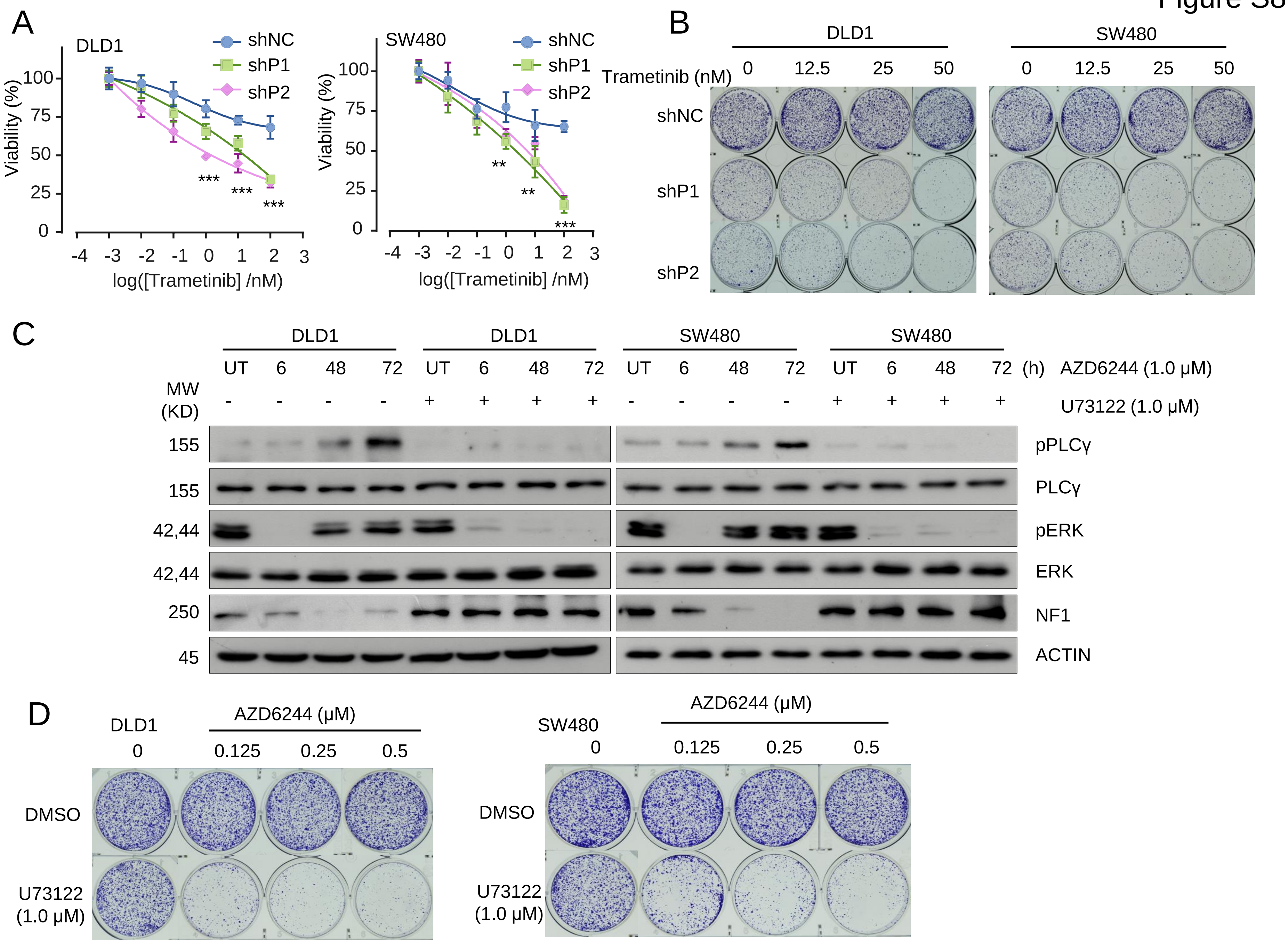

A
DLD1
shNC
shP1
shP2
Viability (%)
100
75
50
25
0
***
***
***
-4 -3 -2 -1 0 1 2 3
log([Trametinib] /nM)
SW480
shNC
shP1
shP2
Viability (%)
100
75
50
25
0
**
**
***
-4 -3 -2 -1 0 1 2 3
log([Trametinib] /nM)
B
DLD1
SW480
Trametinib (nM)
0 12.5 25 50
0 12.5 25 50
shNC
shP1
shP2
C
DLD1
DLD1
SW480
SW480
UT 6 48 72 UT 6 48 72 UT 6 48 72 UT 6 48 72 (h) AZD6244 (1.0 μM)
MW (KD)
- - - - + + + + - - - - + + + + U73122 (1.0 μM)
155 pPLCγ
155 PLCγ
42,44 pERK
42,44 ERK
250 NF1
45 ACTIN
D
DLD1
AZD6244 (μM)
0 0.125 0.25 0.5
DMSO
U73122 (1.0 μM)
SW480
AZD6244 (μM)
0 0.125 0.25 0.5
DMSO
U73122 (1.0 μM)
