# Supplementary material for: PDE4DIP contributes to colorectal cancer growth and chemoresistance through modulation of the NF1/RAS signaling axis
Source: Cell Death Dis. 2023 Jun 24;14(6):373. doi: 10.1038/s41419-023-05885-y (PMC10290635; doi:10.1038/s41419-023-05885-y)

Figure 1

C

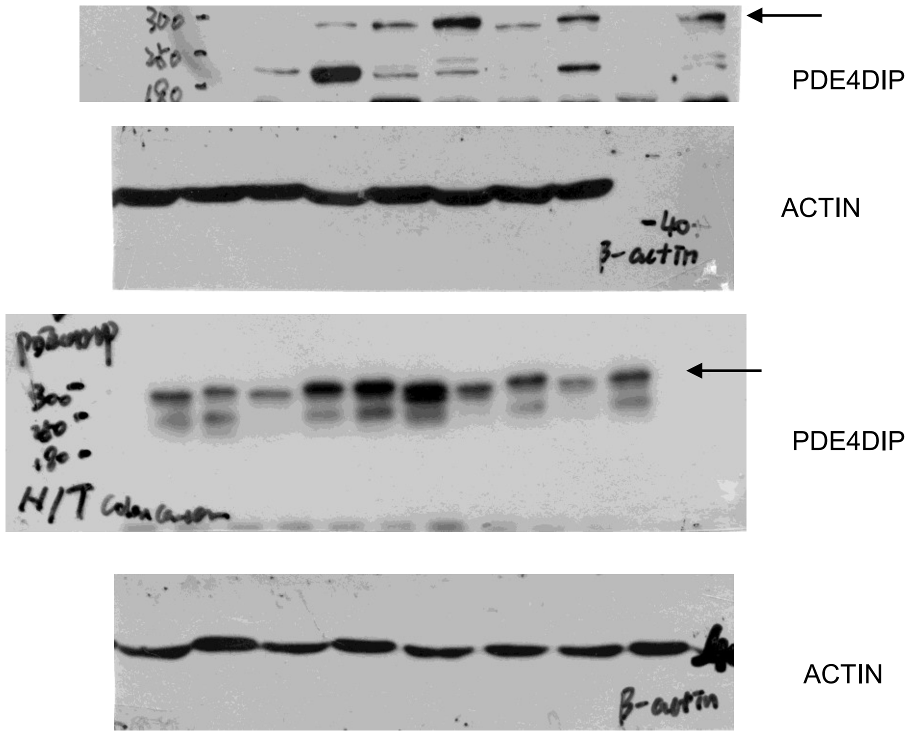


Figure 2

A

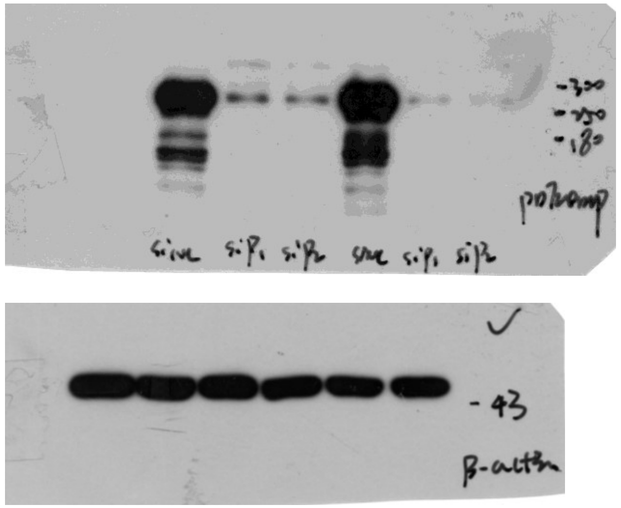


E

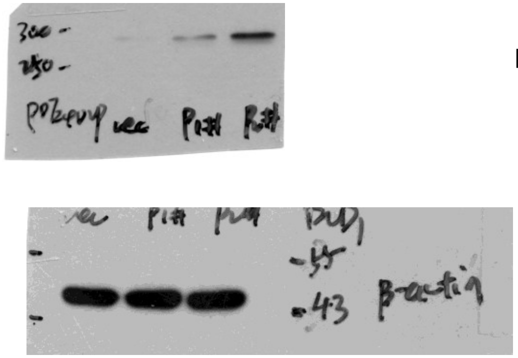

Figure 3

B

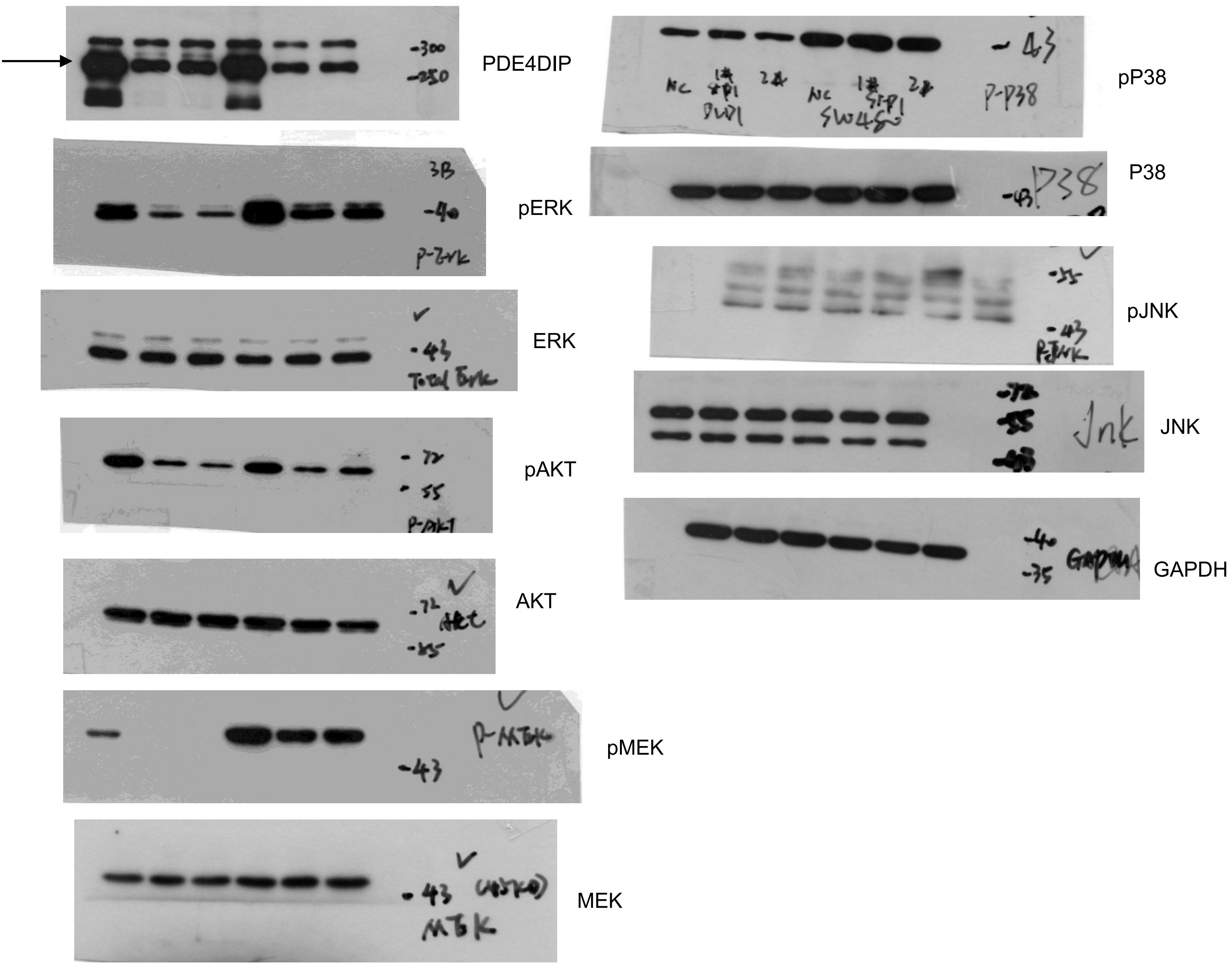
PDE4DIP
pERK
ERK
pAKT
AKT
pMEK
MEK
pP38
P38
pJNK
JNK
GAPDH

C

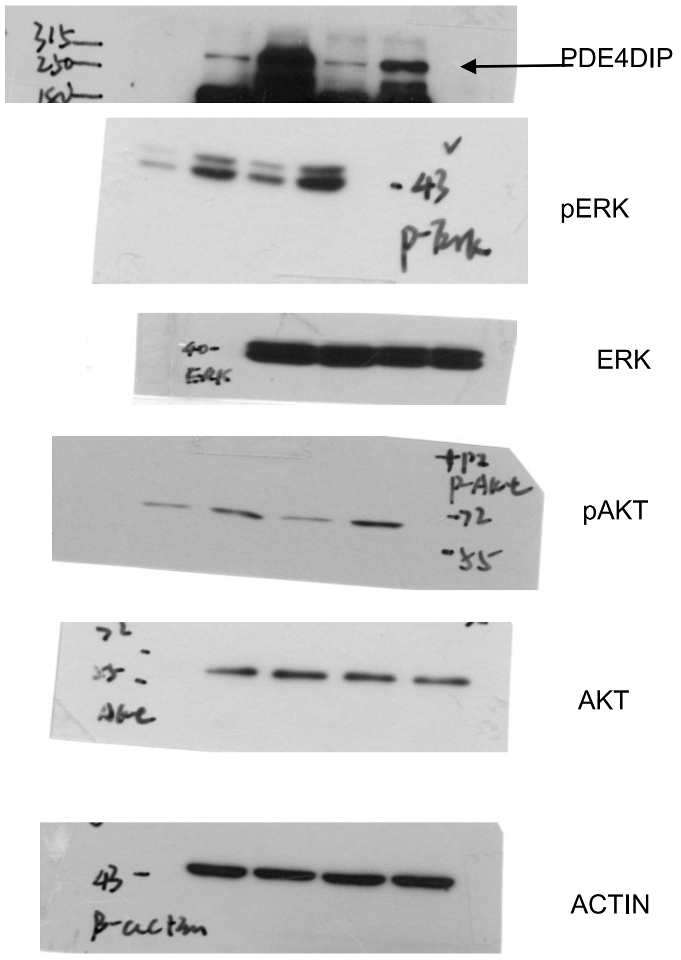
PDE4DIP
pERK
ERK
pAKT
AKT
ACTIN

Figure 3

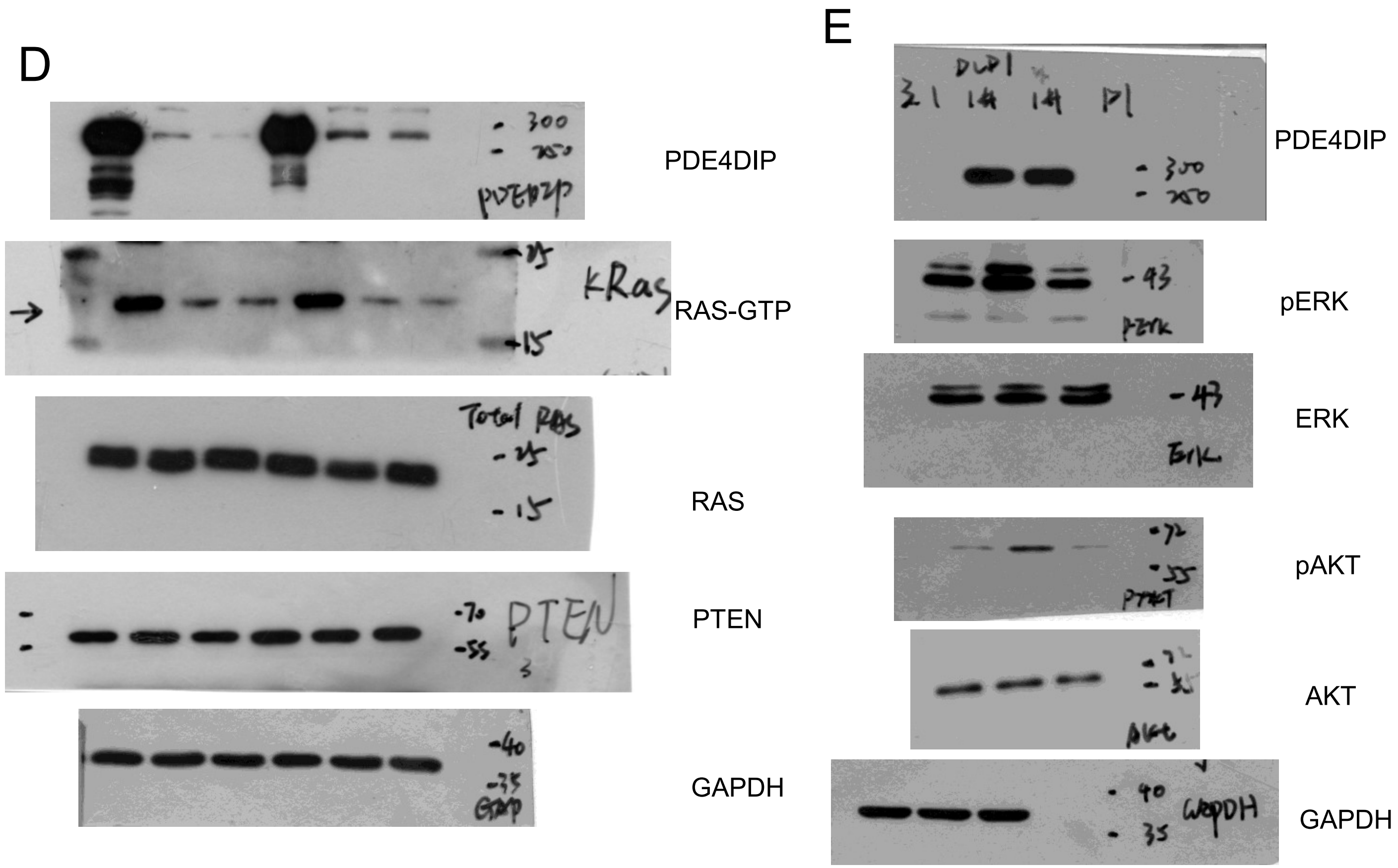
D
PDE4DIP
RAS-GTP
RAS
PTEN
GAPDH
E
PDE4DIP
pERK
ERK
pAKT
AKT
GAPDH

Figure 3

G

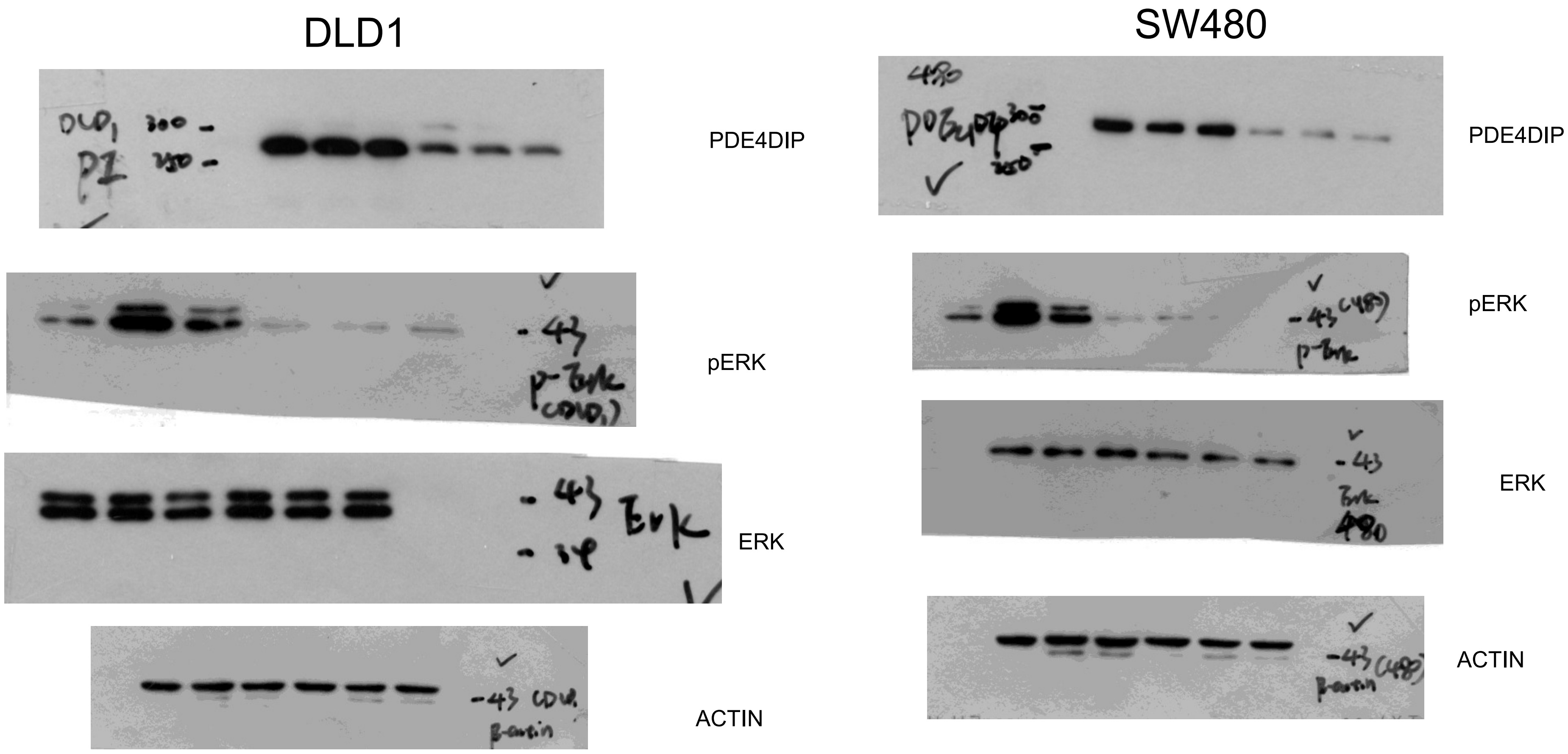

Figure 4

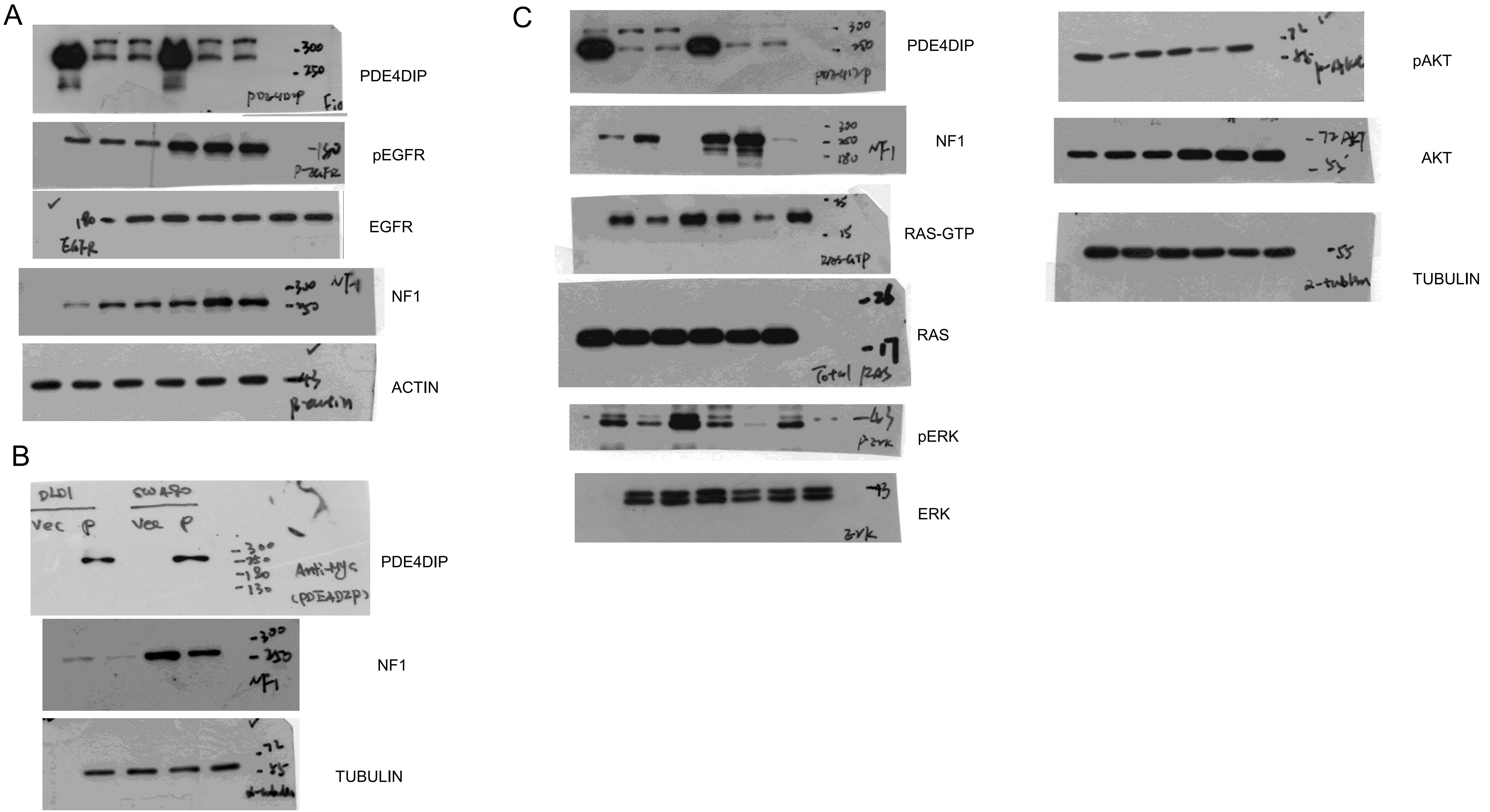
A
PDE4DIP
pEGFR
EGFR
NF1
ACTIN
B
PDE4DIP
NF1
TUBULIN
C
PDE4DIP
NF1
RAS-GTP
RAS
pERK
ERK
pAKT
AKT
TUBULIN

Figure 4

F

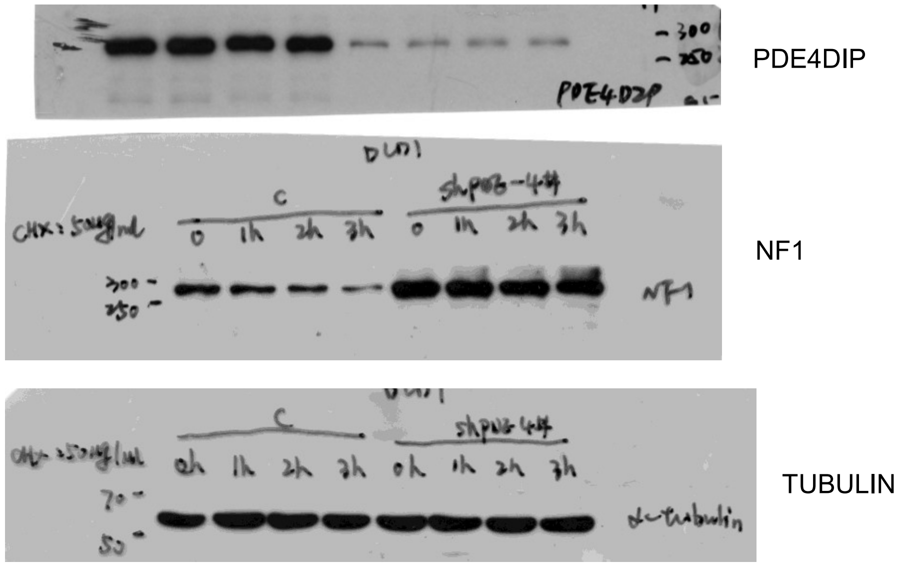


G

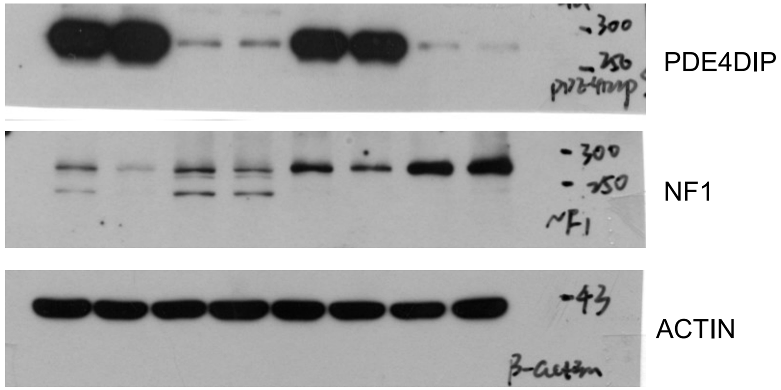


H

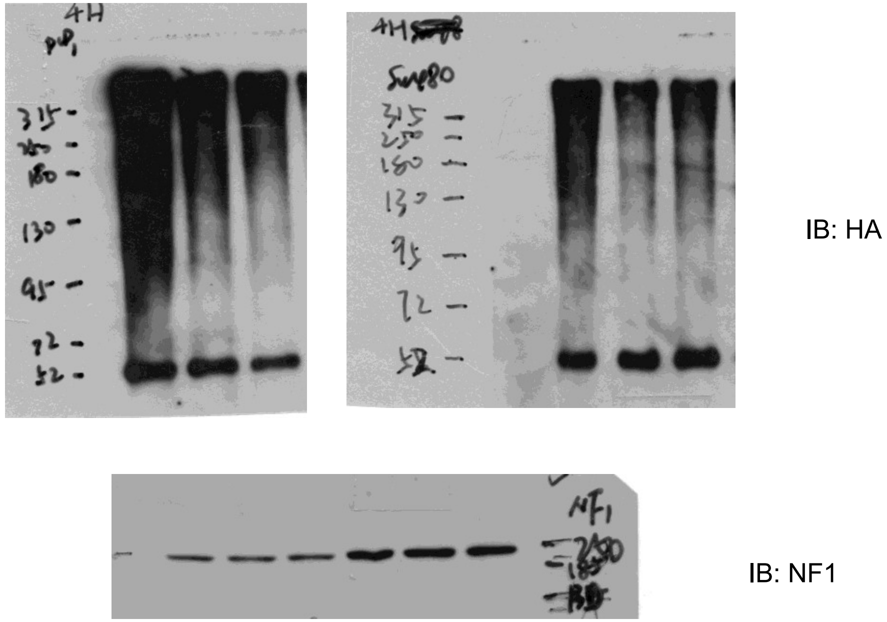

Figure 5

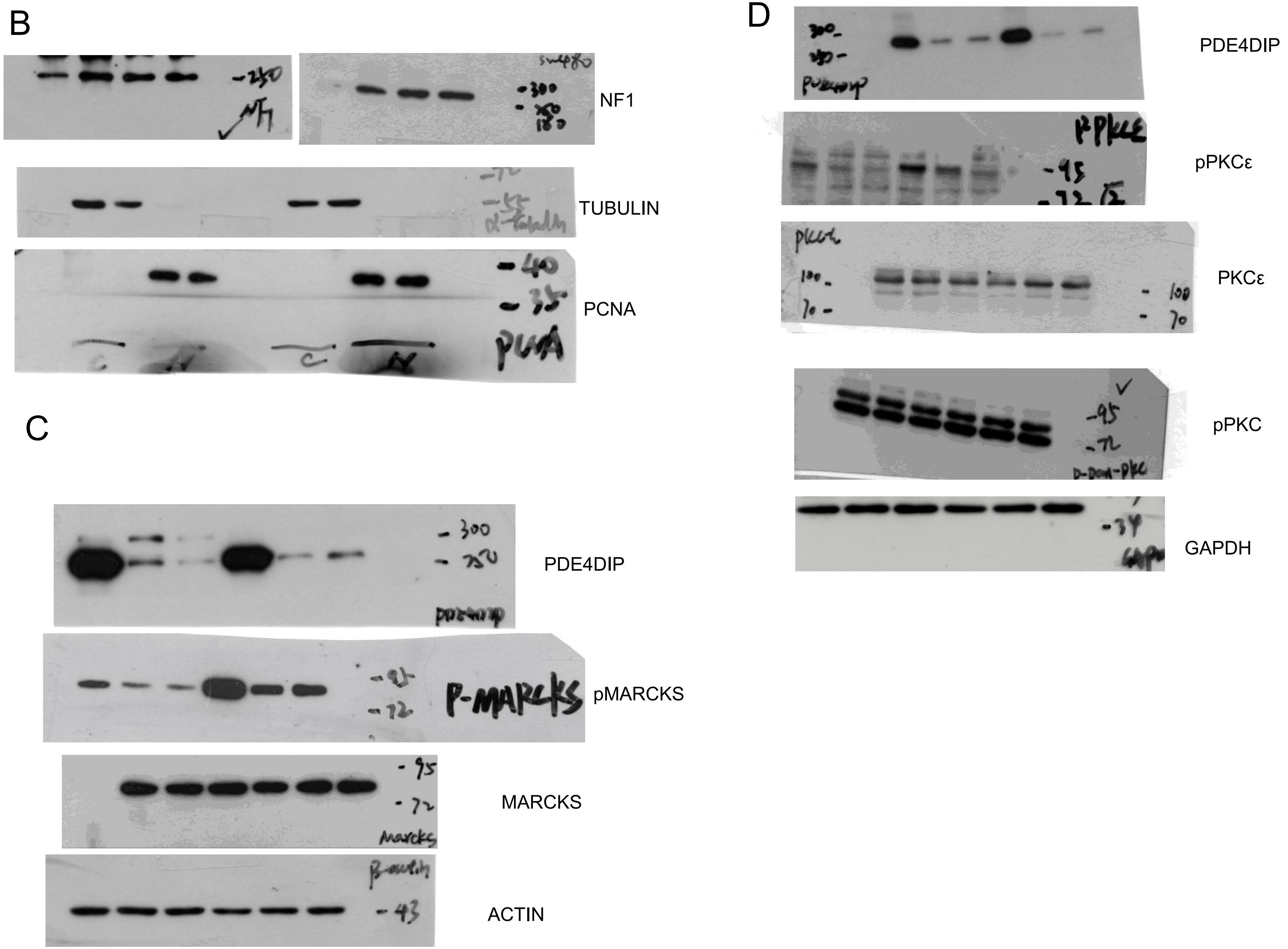
B
NF1
TUBULIN
PCNA
C
PDE4DIP
pMARCKS
MARCKS
ACTIN
D
PDE4DIP
pPKCε
PKCε
pPKC
GAPDH

Figure 5

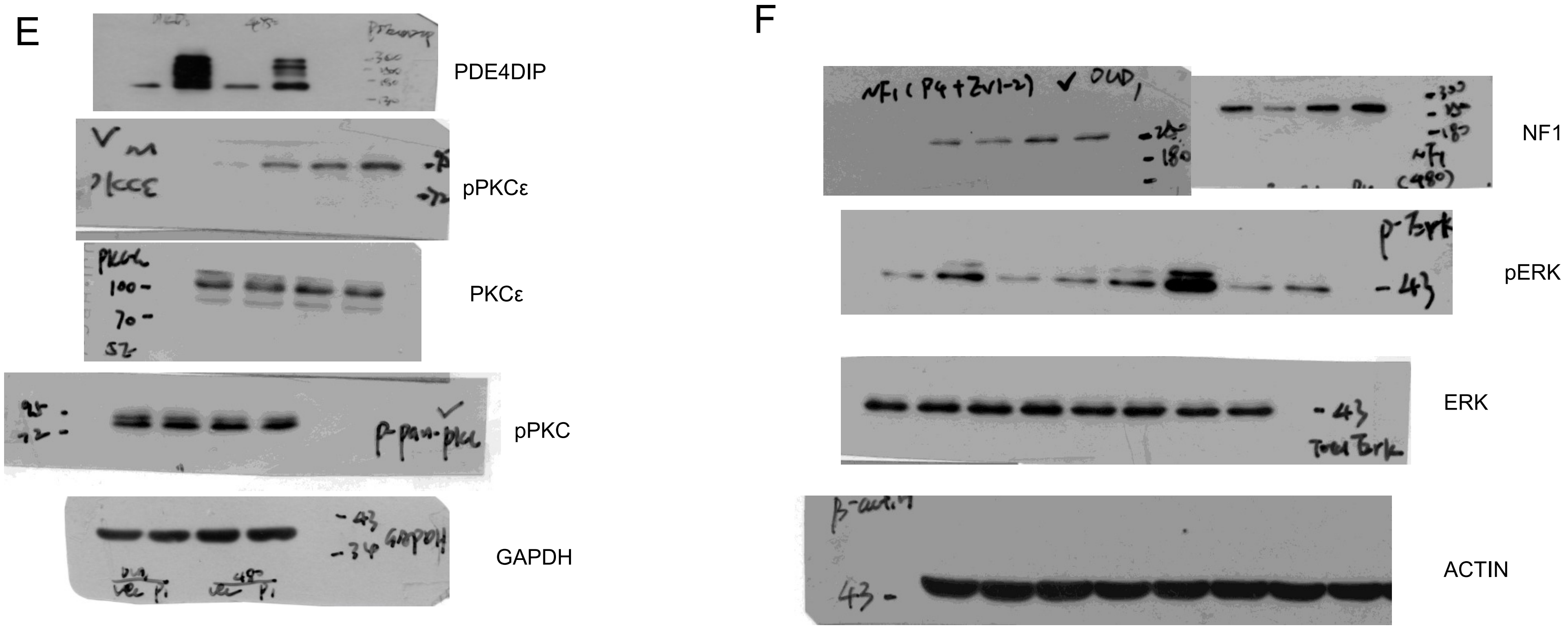

Figure 6

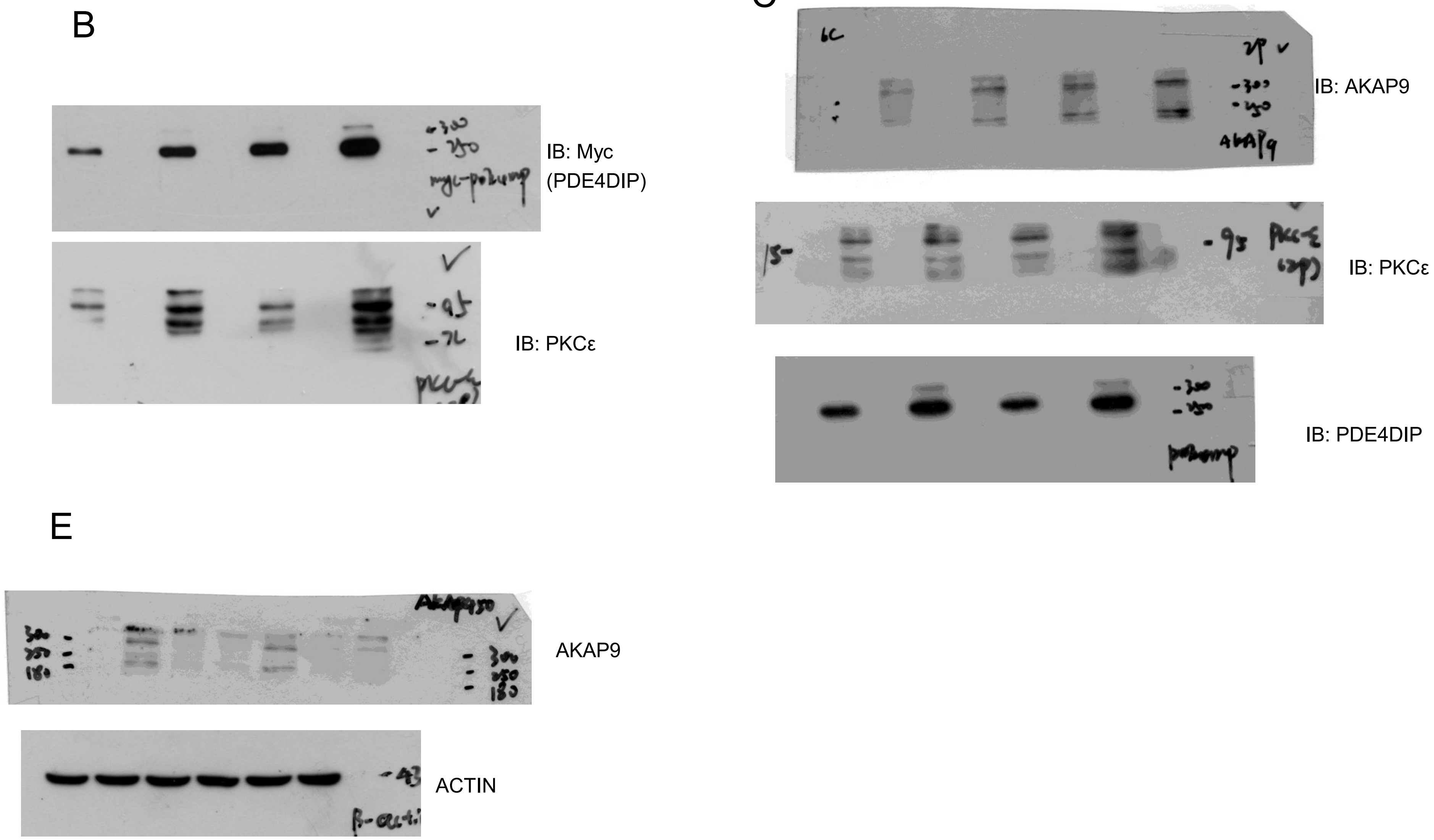
B
IB: Myc
(PDE4DIP)
IB: PKCε
C
IB: AKAP9
IB: PKCε
IB: PDE4DIP
E
AKAP9
ACTIN

Figure 6

F

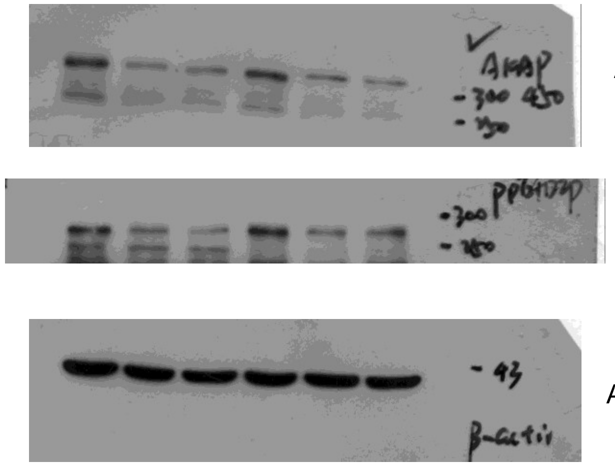
AKAP9
PDE4DIP
ACTIN

H

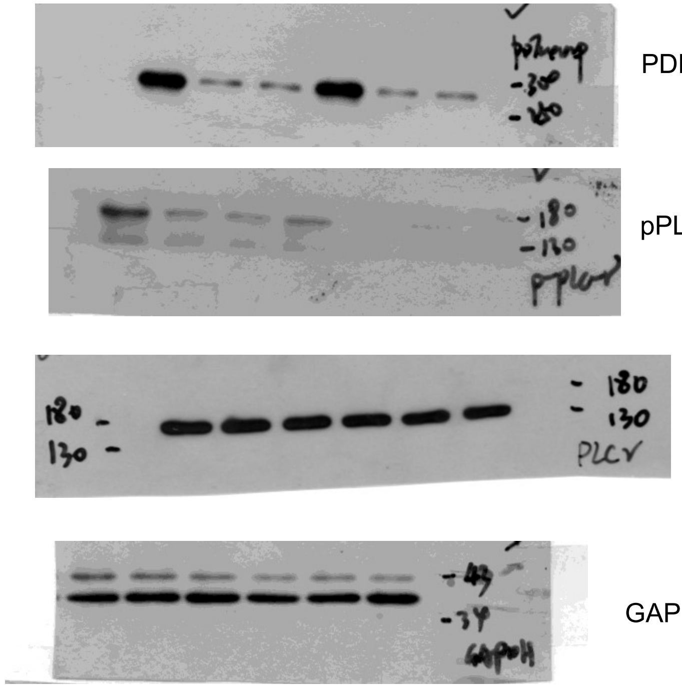
PDE4DIP
pPLCγ
PLCγ
GAPDH

I

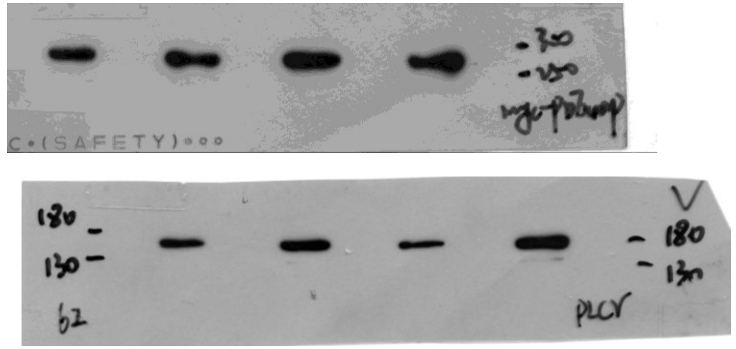
IB: Myc
(PDE4DIP)
IB: PLCγ

Figure 7

C

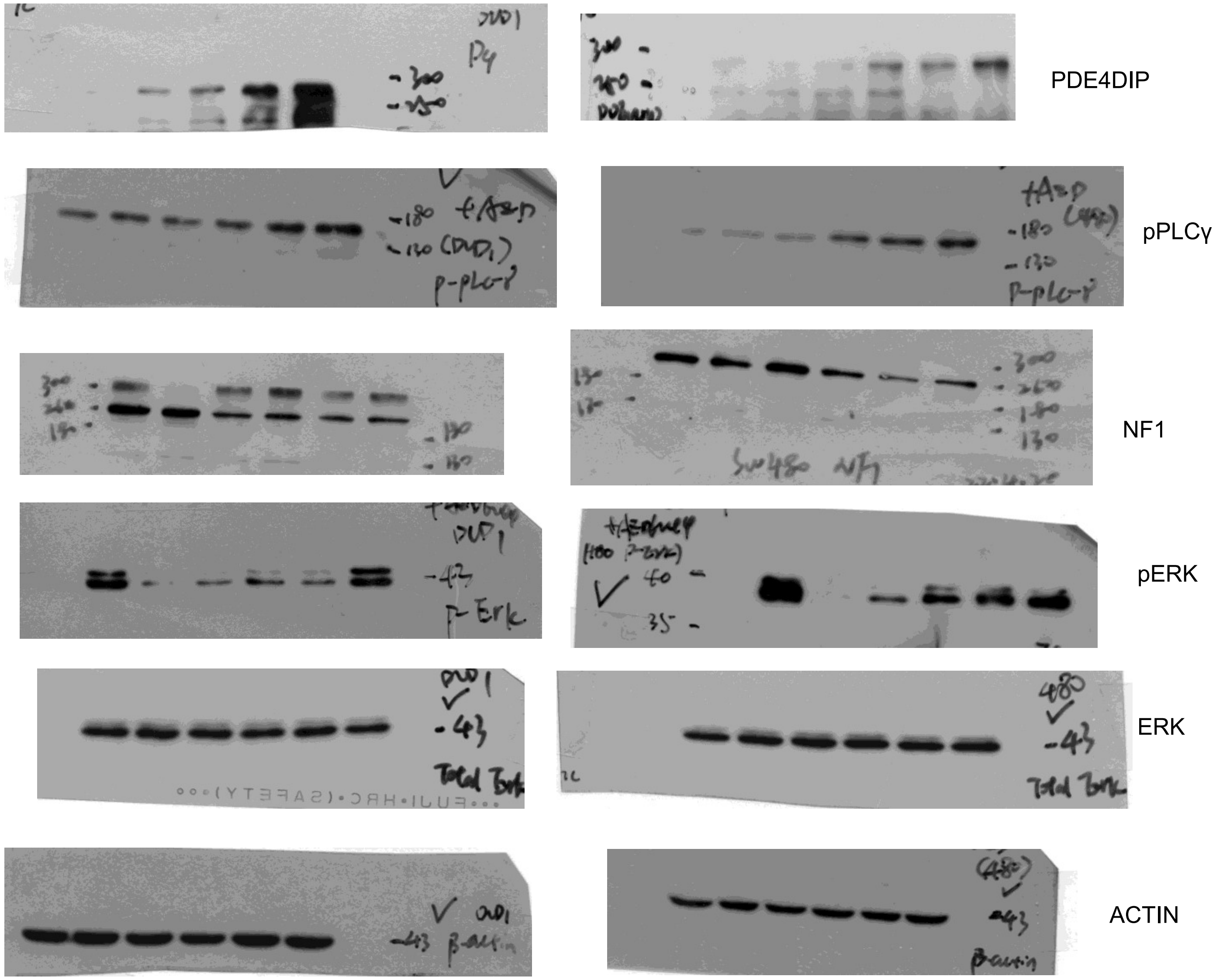

Figure 7

D

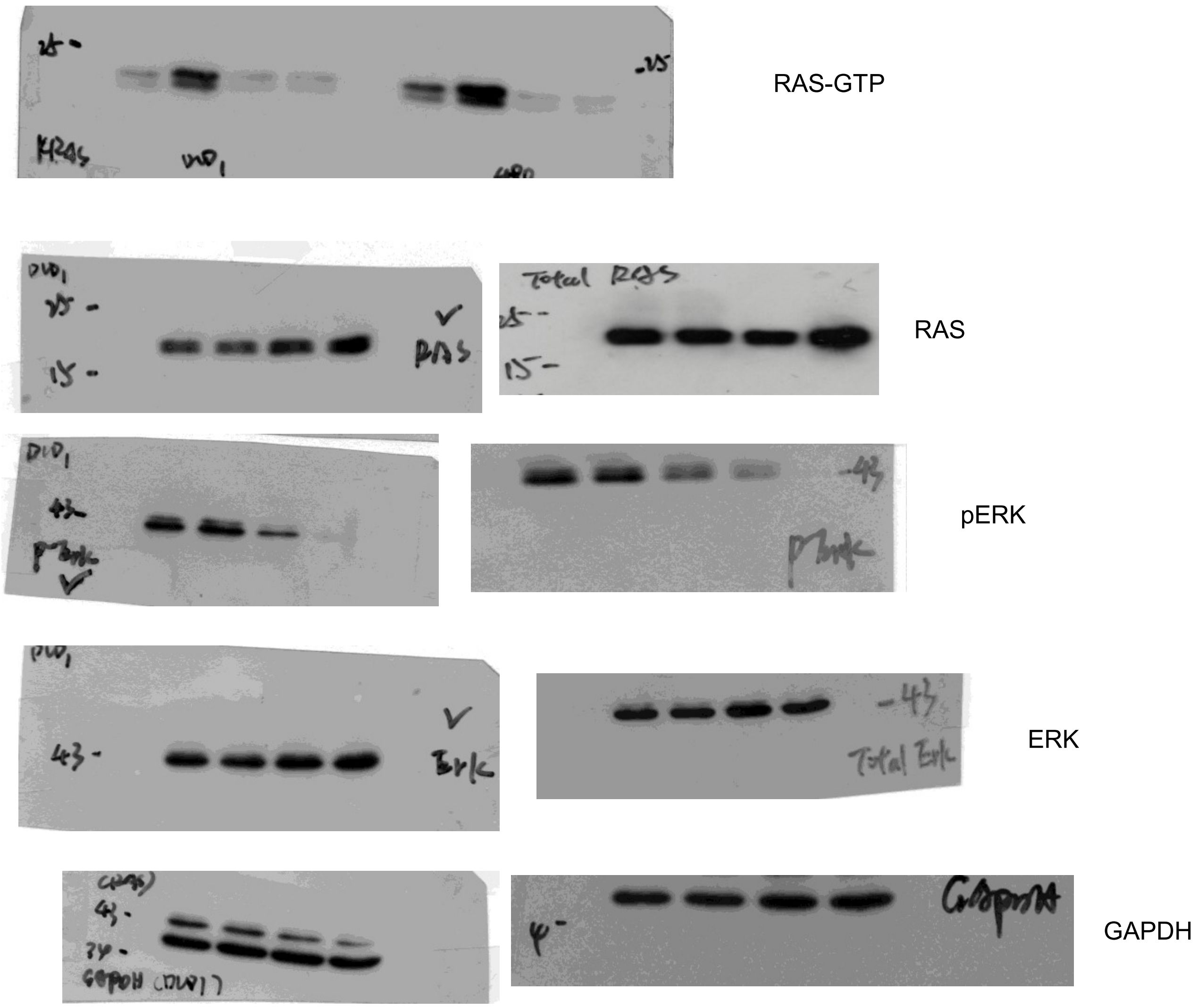

Figure 7

E

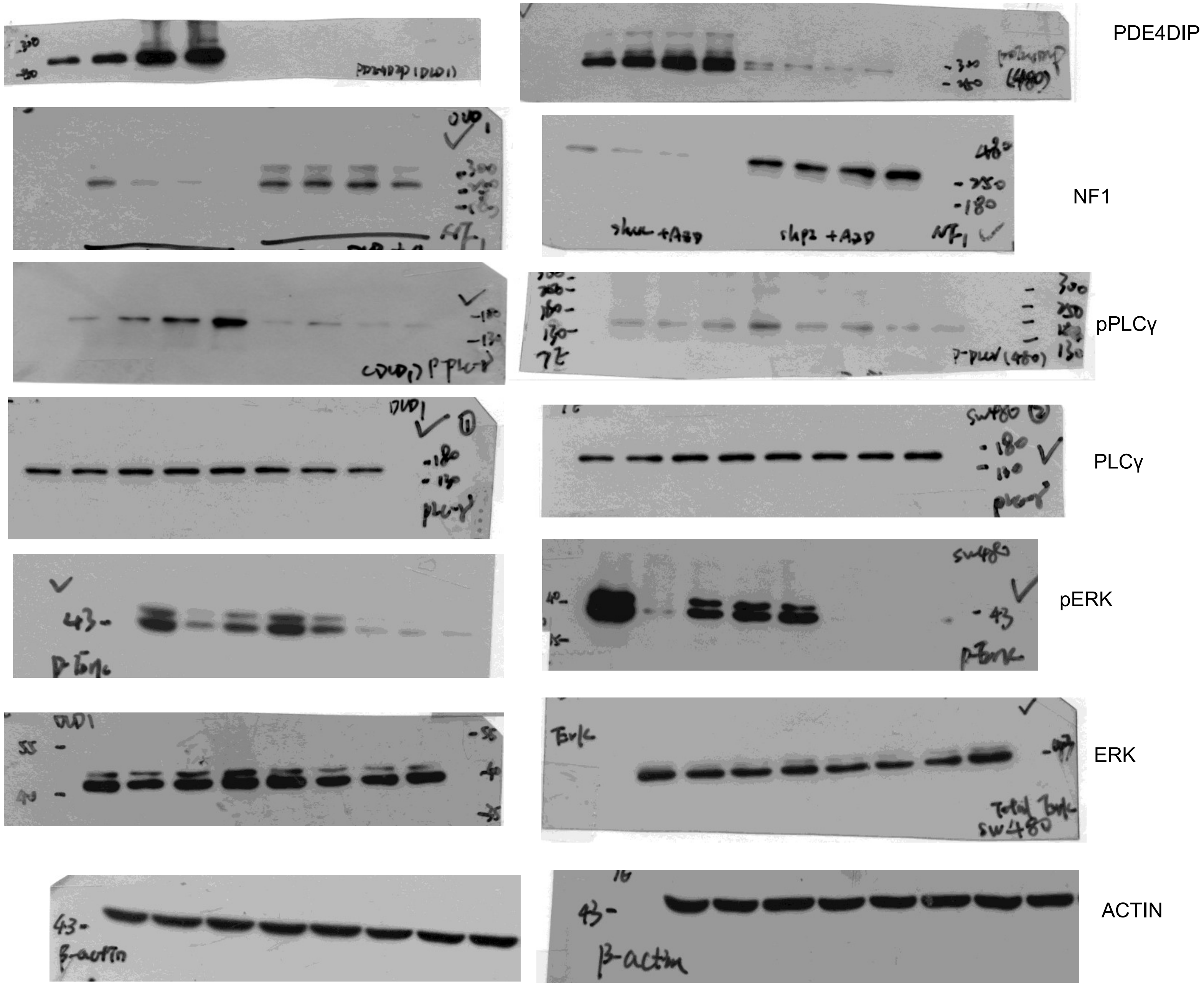

Figure 7

F

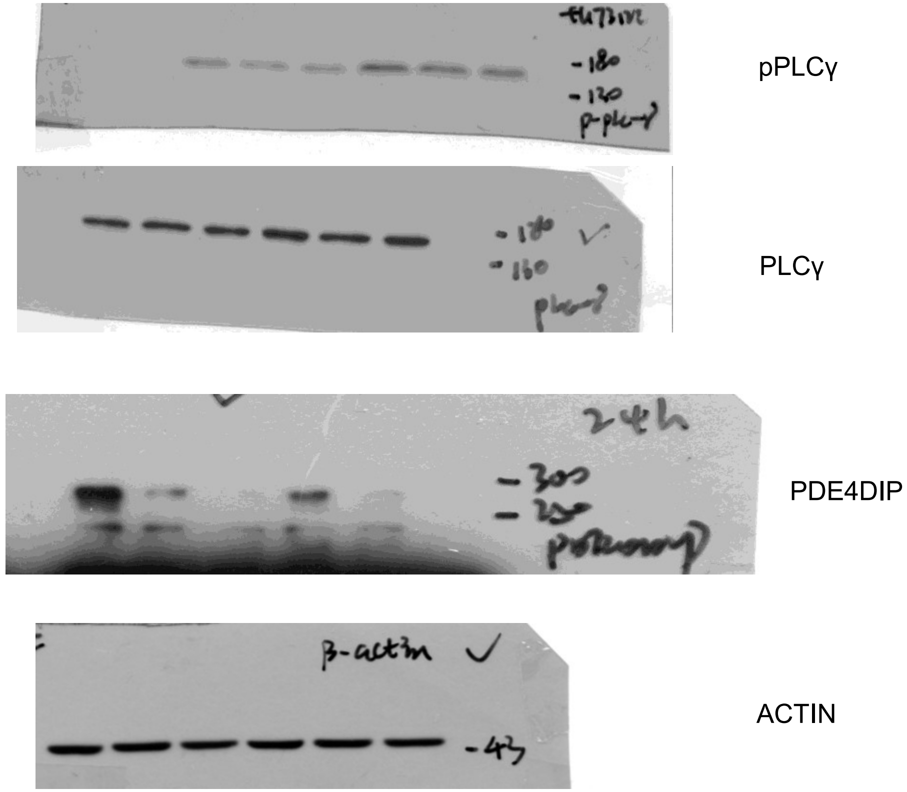


G

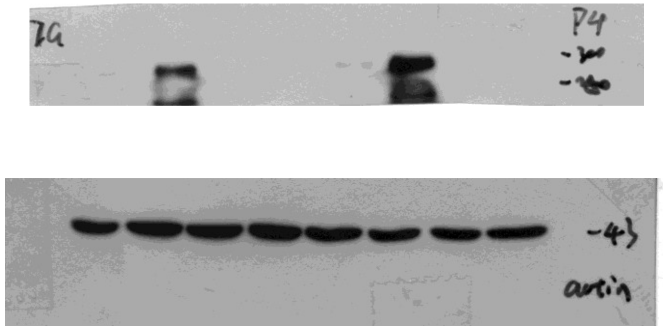

Figure S1

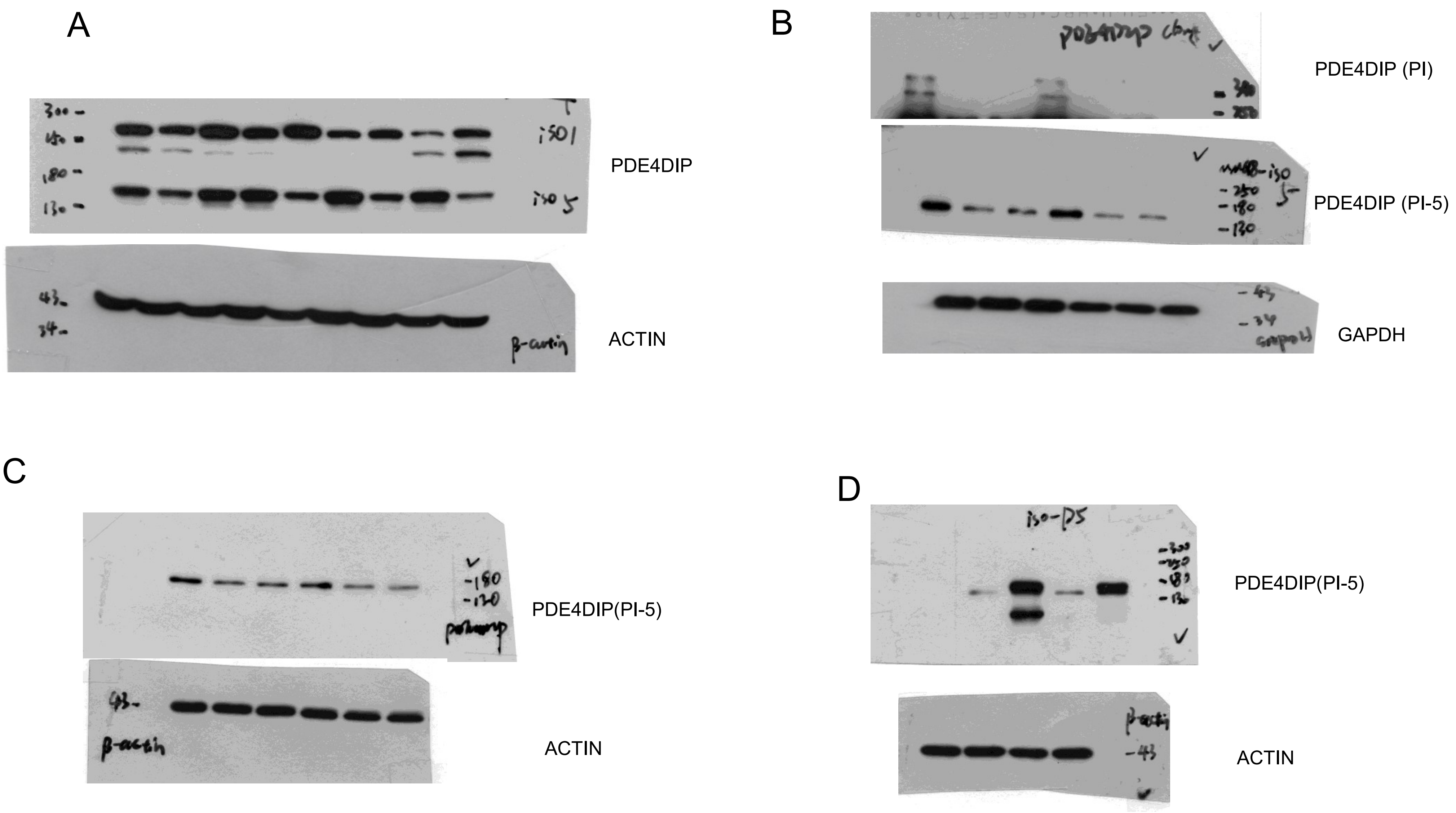
A
PDE4DIP
ACTIN
B
PDE4DIP (PI)
PDE4DIP (PI-5)
GAPDH
C
PDE4DIP(PI-5)
ACTIN
D
PDE4DIP(PI-5)
ACTIN

## Figure S2

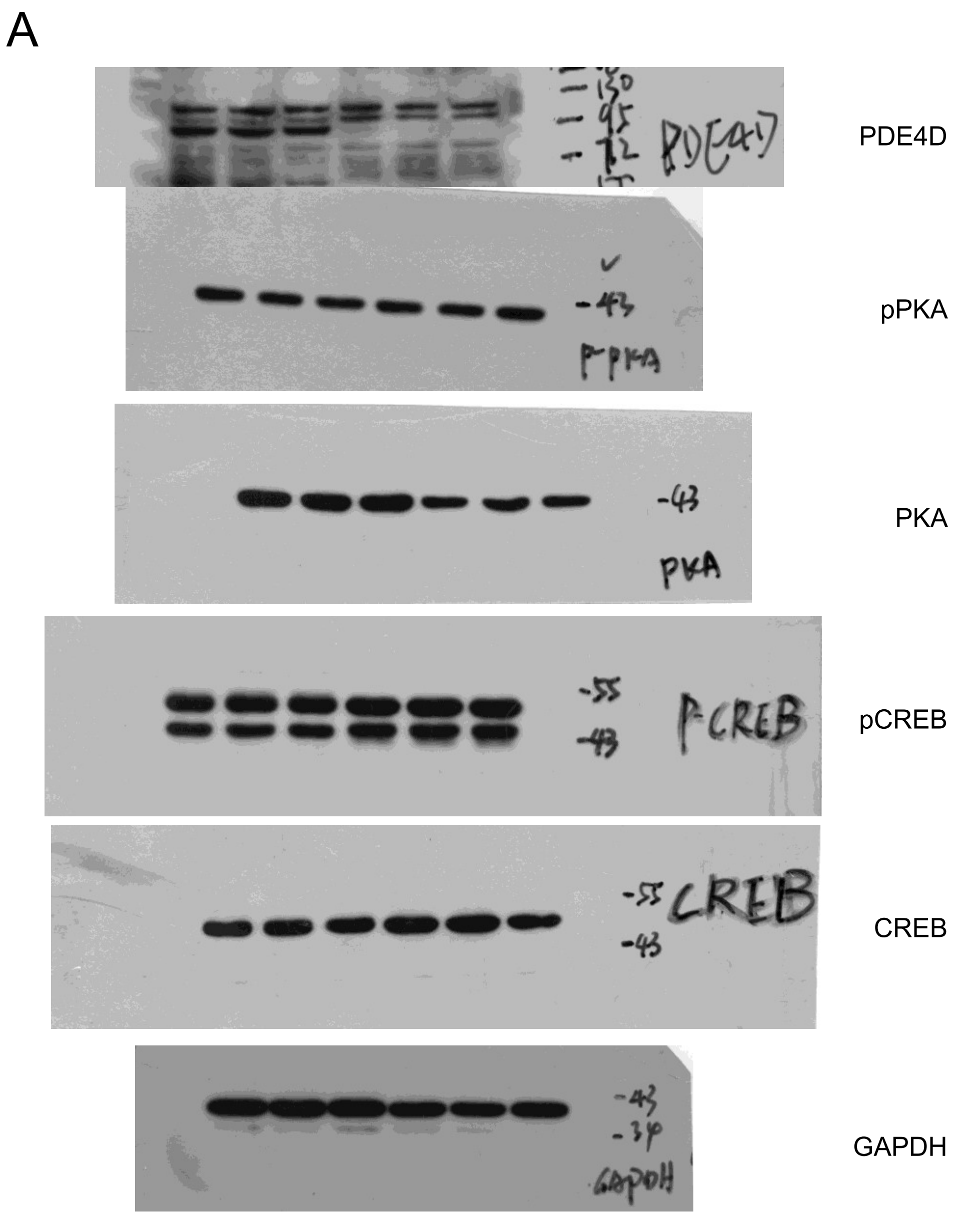


## Figure S4

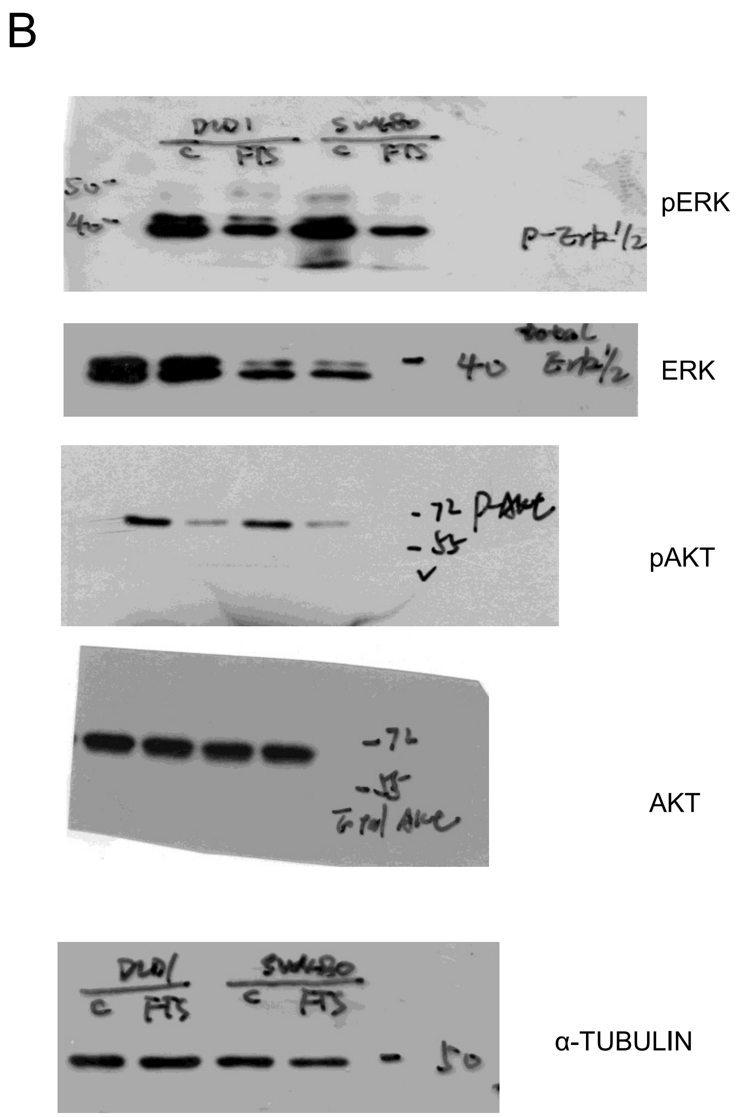

Figure S5

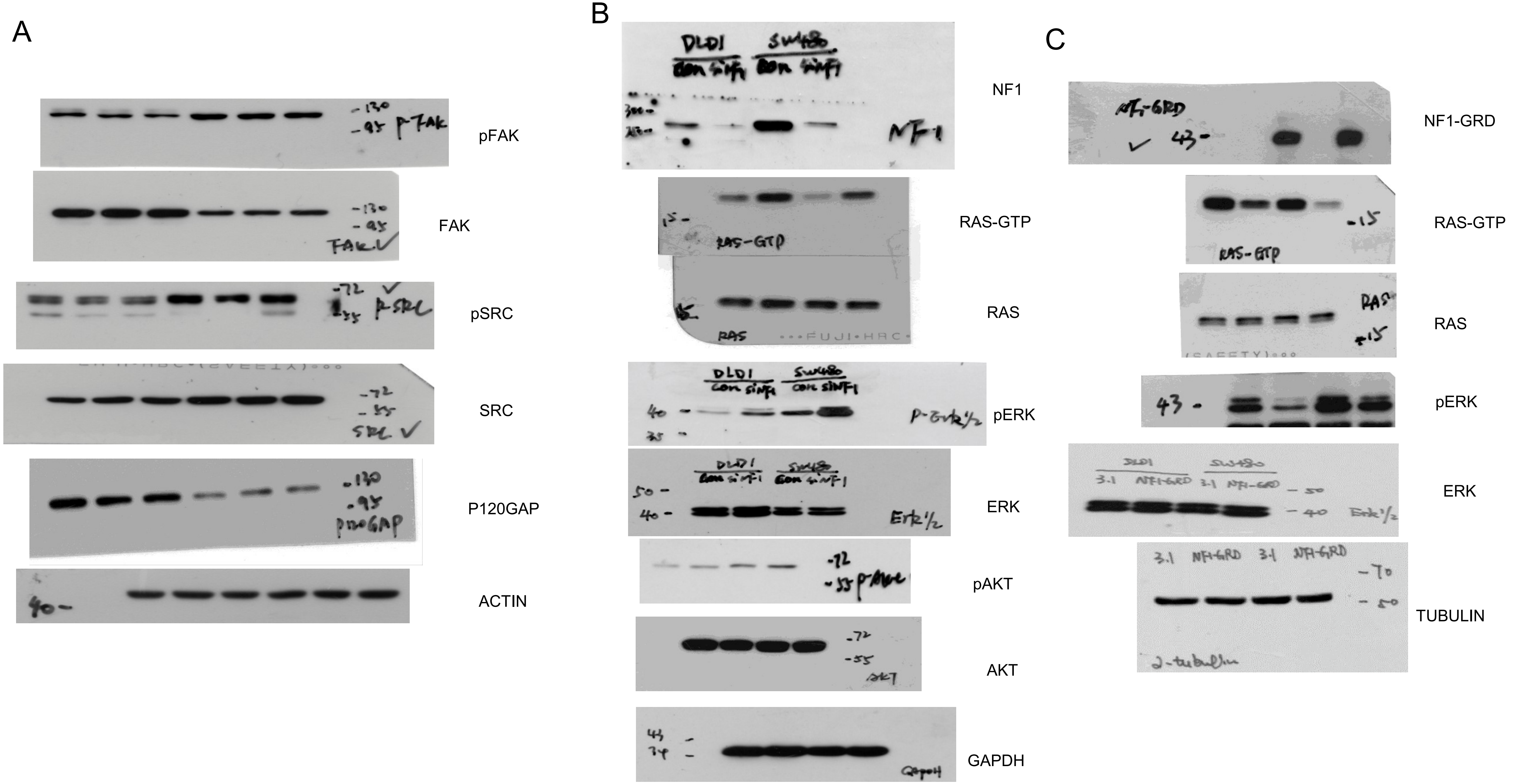
A
pFAK
FAK
pSRC
SRC
P120GAP
ACTIN
B
NF1
RAS-GTP
RAS
pERK
ERK
pAKT
AKT
GAPDH
C
NF1-GRD
RAS-GTP
RAS
pERK
ERK
TUBULIN

Figure S6

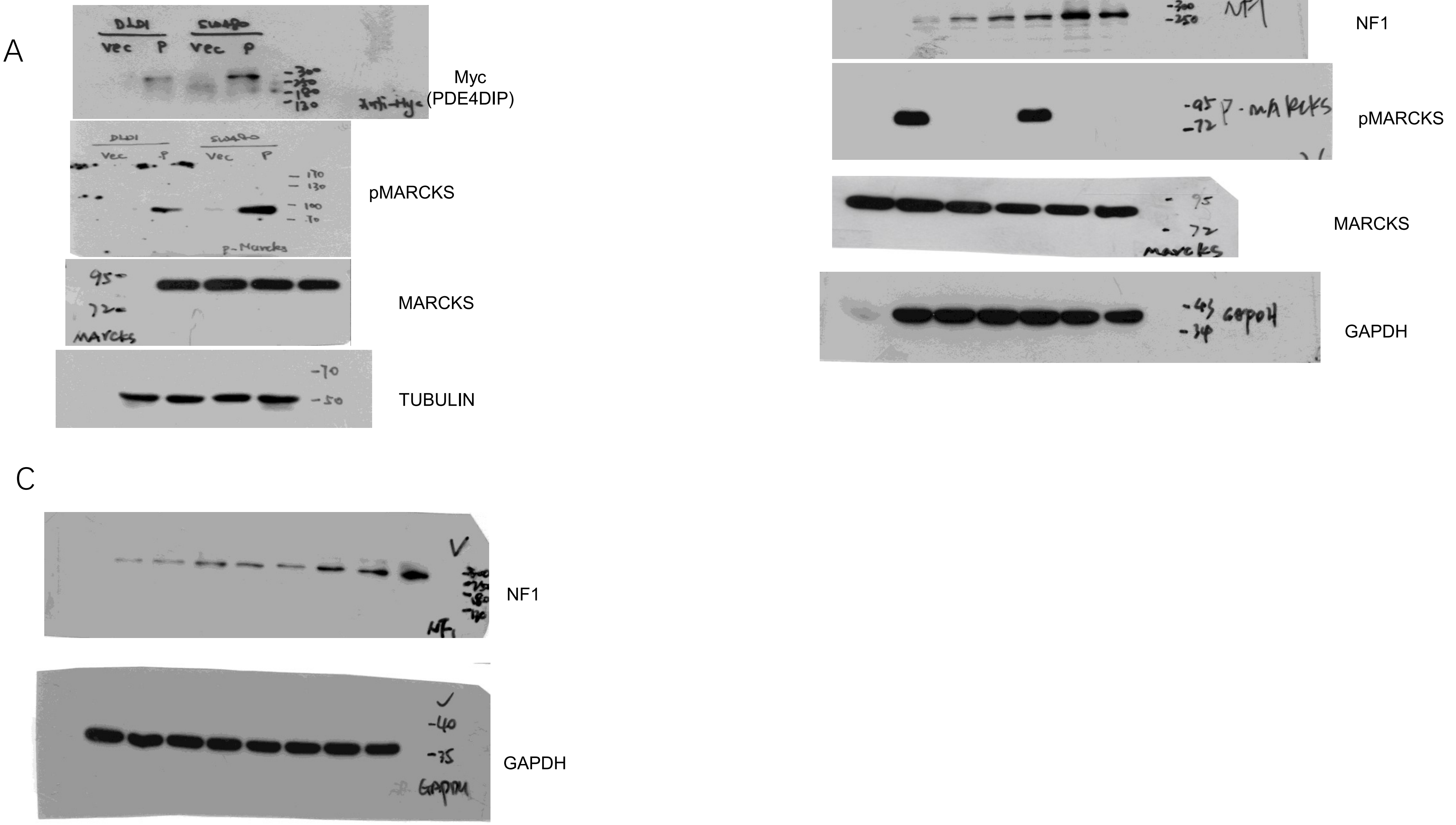
A
Myc (PDE4DIP)
pMARCKS
MARCKS
TUBULIN
B
NF1
pMARCKS
MARCKS
GAPDH
C
NF1
GAPDH

# Figure S6

D

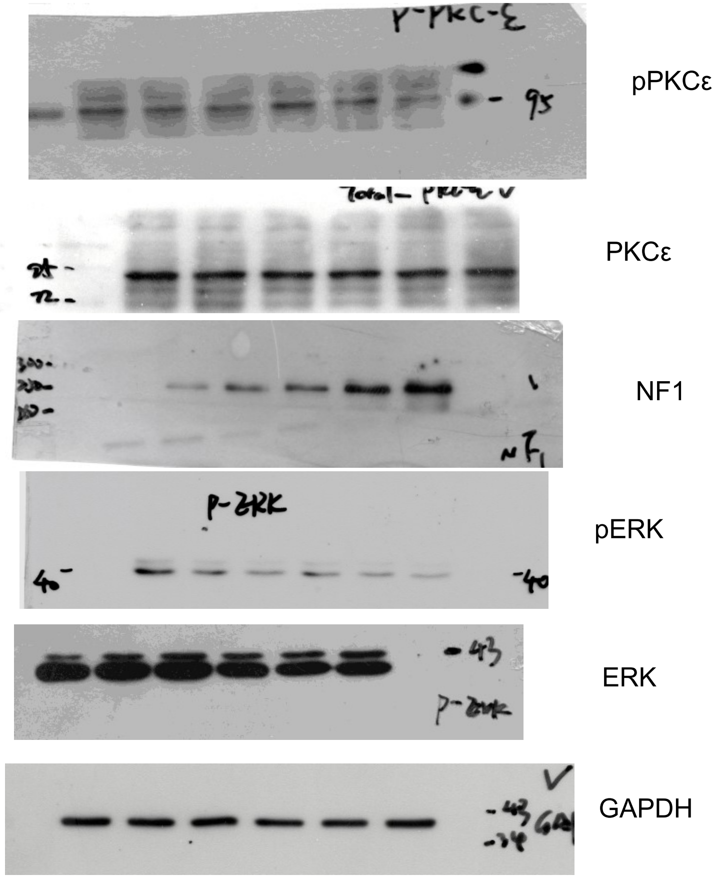


F

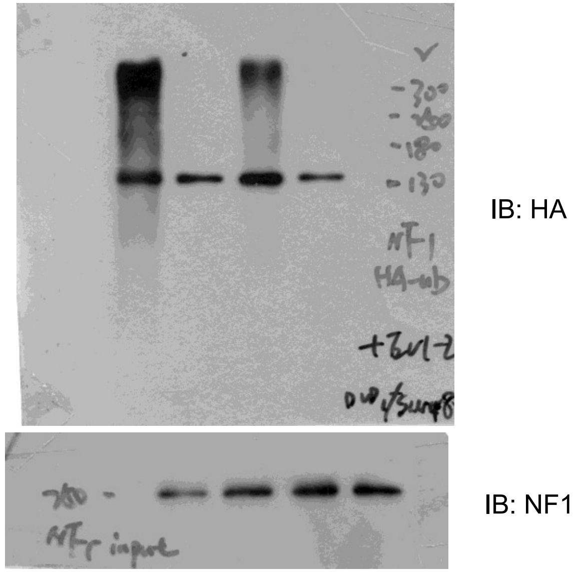


G

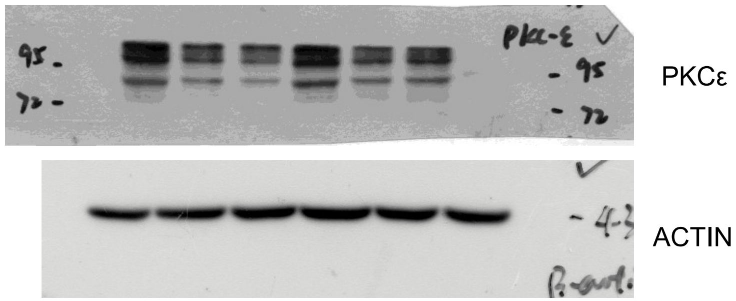

Figure S7

A

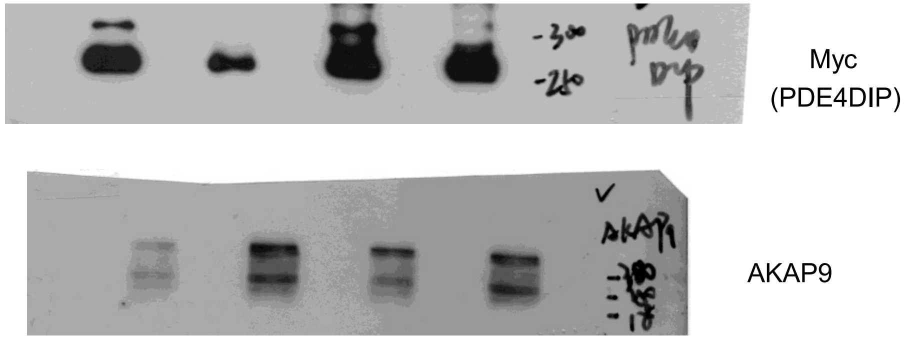

Myc
(PDE4DIP)

AKAP9

B

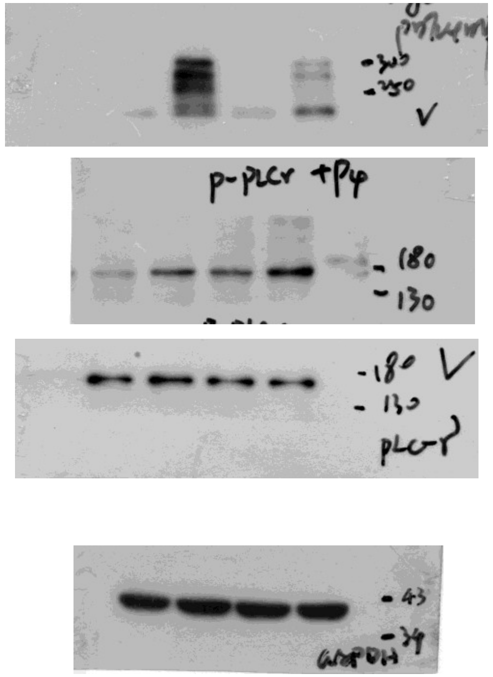

Myc
(PDE4DIP)

pPLCγ

PLCγ

GAPDH

Figure S8

C

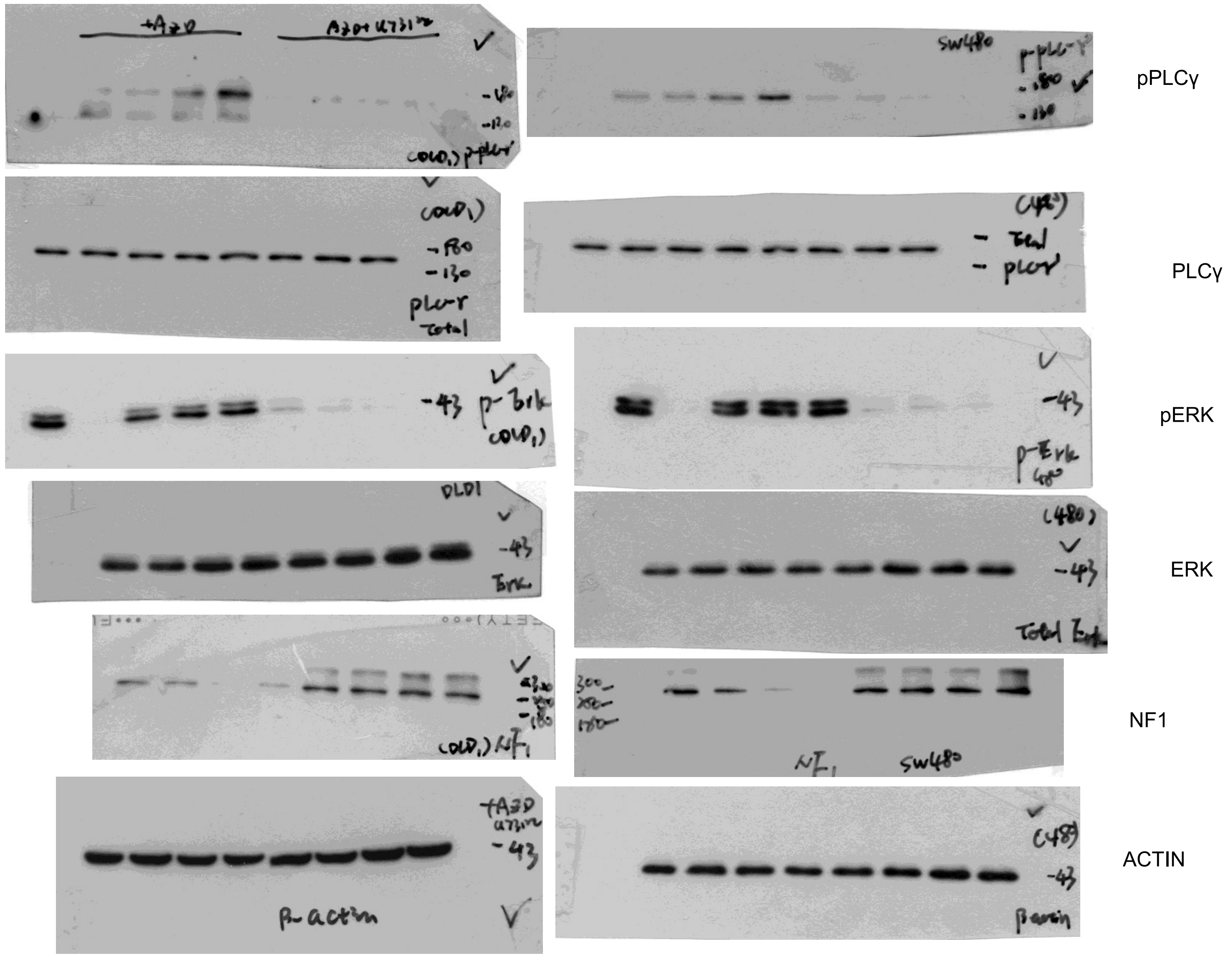

Supplement: Supplementary file 3 — Original Data File [file 41419_2023_5885_MOESM3_ESM.pdf]
